# Impact of pre-chemotherapy food intake on chemotherapy-induced nausea and vomiting in patients with early breast cancer: a subanalysis of the randomized SUCCESS C trial

**DOI:** 10.1007/s00520-026-11437-9

**Published:** 2026-11-13

**Authors:** Henning Schäffler, Manuel Hörner, Lothar Häberle, Chloë Goossens, Kerstin Pfister, Elena Leinert, Kristina Veselinovic, Sara Y. Brucker, Uwe Köhler, Georg Heinrich, Andreas Schneeweiss, Matthias W. Beckmann, Peter A. Fasching, Wolfgang W. Janni, Brigitte Rack, Sabine Heublein, Philipp Ziegler

**Affiliations:** 1 Department of Gynecology and Obstetrics, Universitätsklinikum Erlangen, Comprehensive Cancer Center Erlangen-EMN, Friedrich-Alexander-Universität Erlangen-Nürnberg, Erlangen, Germany; 2 Department of Gynecology and Obstetrics, Ulm University Hospital, Ulm, Germany; 3 Biostatistics Unit, Department of Gynecology and Obstetrics, Universitätsklinikum Erlangen, Erlangen, Germany; 4 Department of Women’s Health, Tuebingen University, Tuebingen, Germany; 5 Department of Gynecology and Obstetrics, Klinikum St. Georg, Leipzig, Germany; 6 Schwerpunktpraxis Der Gynäkologie Und Onkologie Fürstenwalde, Klinikum Offenbach, Fürstenwalde, Spree Germany; 7 Gynecologic Oncology, National Center for Tumor Diseases (NCT), University of Heidelberg and German Cancer Research Center (DKFZ), Heidelberg, Germany

**Keywords:** Chemotherapy-induced nausea and vomiting, CINV, Emesis, Chemotherapy, FEC, TC, Breast cancer, Randomized trial, Eating behaviour

## Abstract

**Purpose:**

Chemotherapy-induced nausea and vomiting (CINV) remains a challenging side effect, significantly impacting patients' quality of life [1–3]. Knowledge of eating behaviour’s influence on CINV is still limited. This study improves the understanding of CINV by investigating the impact of pre-chemotherapy food intake within the randomized Phase III SUCCESS C trial.

**Methods:**

The SUCCESS C trial (NCT00847444), enrolled ,463 women with early-stage, HER2-negative breast cancer, receiving either three cycles of 5-fluorouracil (500 mg/m^2^), epirubicin (100 mg/m^2^), and cyclophosphamide (500 mg/m^2^) followed by three cycles of docetaxel (100 mg/m^2^) every 3 weeks (FEC), or six cycles of docetaxel (75 mg/m^2^) and cyclophosphamide (600 mg/m^2^) (TC). The present analysis included patients with completed CINV diary with hourly documentation over 5 days post-chemotherapy.

**Results:**

CINV diary data during chemotherapy cycles 1–3 were available for 1582 patients, of whom 814 received FEC and 768 TC. Patient characteristics between chemotherapy arms were comparable. For FEC, pre-chemotherapy eating behaviour had a significant impact on nausea-free survival (NFS) in cycle 1 (*p* = 0.04) and on emesis-free survival (EFS) in cycle 2 (*p* = 0.02). For TC, no effect on NFS, but a significant effect on EFS (*p* = 0.04 in cycle 1; *p* < 0.01 in cycle 2), was observed. Median EFS was approximately 120 h for all cycles and treatments.

**Conclusion:**

This analysis demonstrates in both regimens less emesis, and for FEC less nausea, of patients with pre-chemotherapy food intake. The effect was most pronounced during the first 24 h after chemotherapy administration. Thus, eating behaviour influences CINV outcomes and warrants further evaluation.

**Supplementary Information:**

The online version contains supplementary material available at https://doi.org/10.1007/s00520-026-11437-9.

## Introduction

Chemotherapy-induced nausea and vomiting (CINV) remains a deteriorating side effect of chemotherapy. Despite the availability of highly active antiemetic drugs, CINV still leads to a reduction in quality of life, treatment delays, dose reductions, and therapy discontinuation and thereby potentially worsens cancer-related outcomes [4]. With the introduction of antibody-drug conjugates (ADCs), the management of CINV has gained renewed relevance [5]. Different chemotherapy regimens have varying emetogenic potential [6], necessitating tailored management [7]. The regimens included in this analysis are classified by current guidelines as highly emetogenic (FEC) and moderately emetogenic (TC) [7].

Different stages of CINV have been described: acute (occurring within the first 24 h), delayed (occurring between day 2 and day 5), anticipatory (occurring before chemotherapy as a conditioned response due to previously experienced CINV), breakthrough (occurring during the first 5 days after chemotherapy despite antiemetic therapy), and refractory CINV (occurring in later cycles as a result of ineffective antiemetic therapy in prior cycles). Importantly, each of these different stages of CINV requires tailored therapeutic strategies [4]. This publication specifically addresses food intake and its timing relative to chemotherapy administration as a potential risk factor influencing the occurrence of CINV. Evidence regarding the impact of food intake in relation to chemotherapy on the occurrence of nausea and vomiting is limited. A systematic review by Mosa et al. described patient-related risk factors for CINV [8] but identified only a single study addressing food intake in direct temporal relation to chemotherapy administration, in which pre-chemotherapy food consumption was reported as a protective factor [9].

Nutritional counselling and education are often recommended for the prevention and management of CINV. Strategies investigated include dietary patterns [10], food selection and preparation, frequent small meals, energy- and protein-enriched foods, and oral supplements [11]. In a randomized trial with 150 breast cancer patients, three dietary education sessions reduced reflux, anorexia, nausea, constipation, and diarrhoea [12]. Another trial reported less nausea and vomiting and better quality of life with personalized diet plans, though the sample was small (*n* = 19) and pre-chemotherapy meals were not clearly considered [13]. An observational study linked frequent small meals with reduced CINV severity, but without statistical significance [14]. Alcohol consumption and adequate protein and carbohydrate intake were also associated with lower CINV [15], though evidence remains limited and based on small studies [16]. Protein-rich meals have reduced nausea in hyperemesis gravidarum [17], prompting studies in cancer patients. In one trial, protein-rich meals plus ginger during the first three days post-chemotherapy reduced delayed CINV [18], possibly by inhibiting gastric hyperactivity [19]. Higher protein intake correlated with greater symptom reduction, suggesting a dose effect, though the sample size was small (N = 28) and the intervention occurred only post-chemotherapy.

Overall, evidence from randomized interventional trials on nutrition counselling for CINV is limited. Current dietary advice during chemotherapy largely mirrors general dietary guidelines and may be beneficial [10]. As emphasized by ASCO [20], data from large cohorts are lacking to support specific nutrition recommendations during chemotherapy [21]. Given the limited and conflicting evidence in the literature, we aim to investigate the impact of pre-chemotherapy food intake on the day of chemotherapy treatment in a large clinical trial with detailed CINV documentation in patients receiving two different chemotherapy regimens. This simple and easily measurable nutritional factor will be analysed using survival analysis methods, comparing the time to onset of nausea or emesis.

## Materials and methods

### SUCCESS C trial

The SUCCESS C trial (NCT00847444), registration date 2009 Feb 17, enrolled patients with HER2-negative early breast cancer with either evidence of positive axillary lymph nodes (pN1) or high-risk node-negative disease (at least pT > 1 or G3 or age < 36 or TNBC) after primary R0 surgery with an indication for adjuvant chemotherapy [22, 23]. The study was approved by the Heinrich Heine University Düsseldorf Ethics Committee and registered under the EU Clinical Trials Register https://www.clinicaltrialsregister.eu/, identifier: 2008–005453-38. Between February 2009 and August 2011, 3642 pre- and postmenopausal women with newly diagnosed HER2-negative intermediate-risk to high-risk breast cancer were enrolled in 231 study centres across Germany [24]. Written consent was obtained from all participants. Patients were randomized to receive either 3 cycles of 5-fluorouracil (500 mg/m^2^ i.v.)/epirubicin (100 mg/m^2^ i.v.)/cyclophosphamide (500 mg/m^2^ i.v.) (FEC) every 21 days (q21d) followed by 3 cycles of docetaxel (100 mg/m^2^) q21d or 6 cycles of docetaxel (75 mg/m^2^ i.v.)/cyclophosphamide (600 mg/m^2^ i.v.) (TC) q21d. After adjuvant chemotherapy, a second randomization took place for either phone-based individual lifestyle intervention or general lifestyle recommendations in the control arm. The trial aimed to assess anthracycline-free chemotherapy in HER2-negative breast cancer and to evaluate the impact of lifestyle intervention on prognosis.

### Data collection

During adjuvant chemotherapy, CINV was evaluated using a study-specific emesis diary during the first 5 days after receiving chemotherapy that included hourly evaluation of nausea, retching, vomiting, exact intake of antiemetic medication as well as eating and drinking habits. The diary was explained in detail at the start of chemotherapy, handed out on the first day of each cycle and returned on the first day of the following cycle or at the first follow-up visit for the last cycle.

As described in the SUCCESS C study protocol, the recommended antiemetic prophylaxis in the FEC arm (cycles 1–3) consisted of a NK1 antagonist, dexamethasone (12 mg i.v.) and ondansetron (8 mg twice daily p.o. or equivalent) on day 1 (day of chemotherapy), an NK-1 antagonist on day 2–4, and dexamethasone (8 mg p.o. or i.v.) on days 2–3. In the TC arm, recommended antiemetic prophylaxis was dexamethasone (8 mg p.o.) on the day before chemotherapy, dexamethasone (8 mg i.v. or p.o.) on the day of chemotherapy and the day after, and ondansetron (8 mg i.v.) on the day of chemotherapy.

### Data preparation for analysis

#### Emesis diary

The emesis patient diary consisted of 18 questions on health habits and living conditions and a recurring set of two tables and was handed out for six consecutive days: from day of chemotherapy until the fifth day after chemotherapy. In the first table, nausea, retching, vomiting, application of antiemetic medications, eating, and drinking were documented hourly. In the second table, the type of applied antiemetic medications was documented daily. Nausea was documented in three levels: mild, moderate or severe. The emesis patient diary (in German) can be found in the supplement.

Data from the emesis patient diaries were transferred to a database. The weekly consumption of alcoholic beverages (never, 1–2 times weekly, 3–6 times weekly, daily) and the first occurrence of any type of event in the first table were identified. Medication documentation in the second table was classified into categories (aprepitant, dexamethasone, ondansetron or eq., metoclopramide (MCP), and dimenhydrinate).

#### Additional characteristics

From case report forms, the continuous variables age and body mass index (BMI) were extracted. Furthermore, the ordinal variables ECOG (0, 1, 2+), pathological tumour status (pT1, pT2, pT3+), pathological node status (pN0, pN+), and smoking (smoker, non-smoker) were captured. For smoking, the additional category occasional smoker was subordinated to smoker, as the self-attribution of occasional smoking is often ambivalent [25].

#### Endpoints

An emetic episode was defined as the occurrence of retching, vomiting, or the application of rescue medication, where all three documentation levels for retching and vomiting are considered. Emesis-free survival (EFS) was defined as the time in hours from chemotherapy administration to the first emetic episode, or, if none occurred, until 12 PM on the last documented day of the respective chemotherapy cycle.

A nausea episode was defined as the occurrence of an emetic episode or of nausea, where all three documentation levels for nausea were considered. Nausea-free survival (NFS) was defined as time in hours from chemotherapy administration to the first episode of nausea, or, if none occurred, until 12 PM on the last documented day in the respective chemotherapy cycle.

To ensure comparability with present studies on chemotherapy-induced nausea and vomiting, observation time was censored at 120 h (end of period *delayed*).

#### Variable of interest

Pre-chemotherapy food intake was assessed based on the hourly documentation of eating in the patient-reported emesis diary. For each chemotherapy cycle, patients were classified as having “pre-chemotherapy food intake” if any food intake was documented on the day of chemotherapy before the hour of chemotherapy application. Patients without any documented food intake during this period were classified as having “no pre-chemotherapy food intake.” For readability, these groups are subsequently referred to in the text as patients “with food intake” and “without food intake,” respectively. These terms exclusively refer to the above-defined documentation of eating behaviour before chemotherapy and do not imply intentional fasting. Information on the reason for or duration of not eating, as well as the timing, amount, caloric content, or composition of the consumed food, was not collected. Fluid intake was not considered in the definition of pre-chemotherapy food intake.

### Analysis

Patient and tumour characteristics were listed regarding type of chemotherapy (FEC, TC). Eating behaviour before chemo (no pre-chemotherapy food vs. pre-chemotherapy food intake) was listed for different cycles regarding cycle and patient characteristics. Kaplan–Meier plots for EFS and NFS, comparing patients with pre-chemotherapy food intake and patients without pre-chemotherapy food intake, were created for TC and for FEC cycles 1 to 3.

The primary objective was to investigate, whether eating before chemotherapy on the day of chemotherapy administration results in different EFS, respectively NFS, in addition to other well-known prognostic factors. The analysis was performed separately for FEC and TC regimens. For this purpose, for every FEC chemo cycle (three cycles) and for every TC chemo cycle (three cycles), a multiple Cox regression model, the *full model*, was fitted with outcome EFS, respectively NFS, and the different predictors (eating behaviour (categorial; pre-chemotherapy food intake vs. without pre-chemotherapy food intake, patient age (continuous), smoking (categorial; non-smoker, smoker), alcohol per week (categorial; never, 1–2×, and 3–7×), and BMI (continuous)).

Furthermore, for every chemo cycle of FEC and TC another multiple Cox regression model, the *base model*, with the same predictors as the full model, except eating behaviour, was fitted with outcome EFS, respectively NFS. For every chemo type and cycle, full model and base model are compared via the likelihood ratio test (in sum six comparisons for FEC and six comparisons for TC—one per cycle for EFS, one per cycle for NFS). Multiple testing was taken into account through Holm-Bonferroni correction for the P-values for every chemotherapy-endpoint combination separately (FEC for endpoint NFS was corrected, FEC for endpoint EFS was corrected, TC for endpoint NFS was corrected, and TC for endpoint EFS was corrected). If a significant relation was found, eating behaviour would be a relevant predictor for EFS, respectively, NFS, for the considered chemo FEC or TC.

Patients with missing outcome values were excluded. Smoking was imputed by the most frequent level non-smoker. Other predictors were complete or had no dominant level, thus no further imputation was performed. The proportional hazard assumption was checked for every predictor using the Grambsch–Therneau method [26, 27]. If a predictor violated the assumption, it was considered as stratification factor within the Cox regression model. Some chemo cycles were documented, but missed the time of chemotherapy application. If the patient had documented times in other chemo cycles, the time was imputed by the median of this application times. Adjusted overall HRs for chemotherapy type were estimated. As a sensitivity analysis, corresponding unadjusted HRs for EFS and NFS were estimated using a Cox regression model with chemotherapy type as the sole predictor.

Tests were two-sided with 5% significance level. Data were prepared with tSQL on Microsoft SQL Server Enterprise (64-bit, Version 14.0.3485.1). Calculations were performed with R statistical computing (version 4.2.3, *Shortstop Beagle*) within the RStudio environment.

## Results

### Patients

**Fig. 1:**
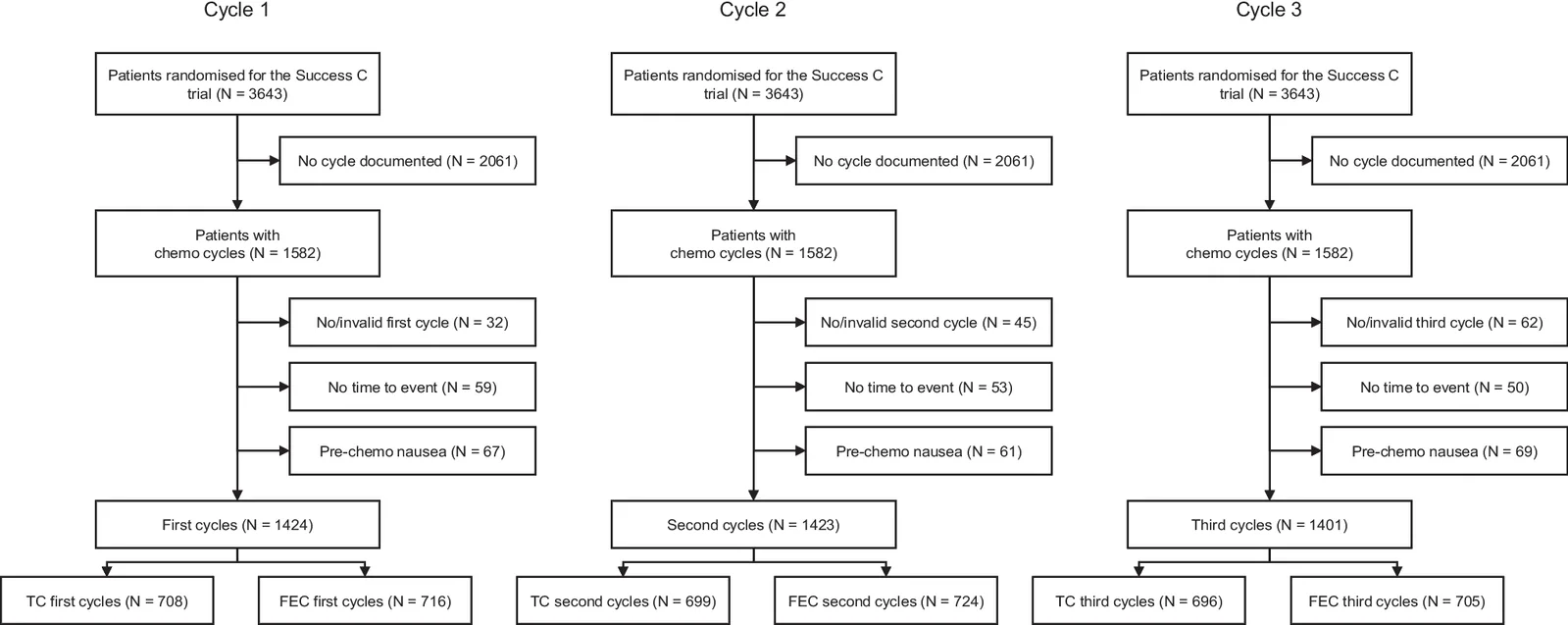
Patient flowchart for cycles 1–3

Of the 3643 patients enrolled in the SUCCESS C trial, 2061 patients had to be excluded due to missing documentation of chemotherapy cycles, leaving 1582 patients for this analysis. Further exclusion reasons were invalid cycle documentation, missing documentation regarding events, or a pre-chemo nausea event resulting in 1424, 1423, and 1401 patients for cycles 1, 2, and 3, respectively (Fig. 1).

Of the 814 patients that received FEC, the mean age was 54.6 years and the mean BMI was 26.4 kg/m^2^. Furthermore, 44.8% reported never consuming alcohol and 89.6% were non-smokers. The majority of patients had an ECOG performance status of 0 (71.7%) and had a luminal A-like tumour (54.1%). The most common pathological tumour stage was pT2 (49.1%) and 57.4% of the patients had positive lymph nodes. Patients receiving TC (*N* = 768) had a mean age of 55.2 years and a mean BMI of 26.7 kg/m^2^. In this group, 44.4% of patients reported never consuming alcohol, and 89.4% were non-smokers. 71.3% of patients had an ECOG performance status of 0, 53.1% had a luminal A-like tumour and most of the patients also had a T2 pathological tumour stage (50.1%) and positive lymph nodes (59.6%). Overall, patient characteristics were comparable between chemotherapy groups; for further details, see Table 1.

**Table 1:** Patient and tumour characteristics for all patients with at least one chemo cycle (1582 patients), showing mean and standard deviation or frequency and percentage

Characteristic		Chemotherapy		Total (*N* = 1582)
		FEC (*N* = 814)	TC (*N* = 768)	
Age	Mean (SD)	54.6 (10.1)	55.2 (10.1)	54.9 (10.1)
	Age < 50	258 (31.7%)	247 (32.2%)	505 (31.9%)
	50 ≤ age < 65	408 (50.1%)	368 (47.9%)	776 (49.1%)
	Age ≥ 65	148 (18.2%)	153 (19.9%)	301 (19.0%)
Alcohol per week	Never	342 (44.8%)	315 (44.4%)	657 (44.6%)
	1–2×	339 (44.4%)	306 (43.2%)	645 (43.8%)
	3–7×	82 (10.7%)	88 (12.4%)	170 (11.5%)
	NA	51	59	110
Smoking	Non-smoker	665 (89.6%)	638 (89.4%)	1303 (89.5%)
	Smoker	77 (10.4%)	76 (10.6%)	153 (10.5%)
	NA	72	54	126
Body mass index	Mean (SD)	26.4 (5.2)	26.7 (5.2)	26.5 (5.2)
Pre-cycle emetic episode	No	380 (47.7%)	482 (64.4%)	862 (55.8%)
	Yes	417 (52.3%)	266 (35.6%)	683 (44.2%)
	NA	17	20	37
Pre-cycle nausea episode	No	129 (16.2%)	243 (32.5%)	372 (24.1%)
	Yes	668 (83.8%)	505 (67.5%)	1173 (75.9%)
	NA	17	20	37
Prophylactic antiemetic medication	Aprepitant	198 (24.3%)	62 (8.1%)	260 (16.4%)
	Dexamethason	220 (27.0%)	232 (30.2%)	452 (28.6%)
	Ondansetron or eq	167 (20.5%)	100 (13.0%)	267 (16.9%)
ECOG	0	522 (71.7%)	499 (71.3%)	1021 (71.5%)
	1	199 (27.3%)	193 (27.6%)	392 (27.5%)
	2 +	7 (1.0%)	8 (1.1%)	15 (1.1%)
	NA	86	68	154
Molecular subtype	Luminal A	440 (54.1%)	408 (53.1%)	848 (53.6%)
	Luminal B	172 (21.1%)	172 (22.4%)	344 (21.7%)
	TNBC	202 (24.8%)	188 (24.5%)	390 (24.7%)
Pathological tumour status	pT1	358 (44.0%)	337 (43.9%)	695 (43.9%)
	pT2	400 (49.1%)	385 (50.1%)	785 (49.6%)
	pT3 +	56 (6.9%)	46 (6.0%)	102 (6.4%)
Pathological node status	pN0	347 (42.6%)	310 (40.4%)	657 (41.5%)
	pN +	467 (57.4%)	458 (59.6%)	925 (58.5%)

### NFS and EFS for TC dependent on pre-cycle meal

For TC median observation times for NFS were 69.5 (95% CI (15, 120)), 94 (95% CI (21, 120)), and 120 (95% CI (23, 120)) hours and for EFS were 120 (95% CI (104.75, 120)), 120 (95% CI (120, 120)), and 120 (95% CI (120, 120)) hours in cycles 1, 2, and 3, respectively. No significant effect of pre-chemotherapy eating behaviour on NFS was observed (Likelihood ratio test). However, eating behaviour significantly affected EFS (*P* = 0.04 in cycle 1, *P* < 0.01 in cycle 2, Likelihood ratio test).

Hazard ratios (HRs) for NFS and EFS comparing pre-chemotherapy food intake versus no pre-chemotherapy food intake are shown in Table 2 for all three cycles. For NFS, HR confidence intervals consisted of values below and above one. For EFS, HR in cycle 1 was 0.69 (95% CI (0.50, 0.95)), and HR in cycle 2 was 0.56 (95% CI (0.41, 0.77)). In cycle 3, the 95% CI consisted of values below and above one.

**Table 2:** Cox regression analyses, showing unadjusted and adjusted hazard ratios for pre-chemotherapy food intake vs. without pre-chemotherapy food intake

Chemo	Cycle	Eating behaviour	Nausea-free survival		Emesis-free survival	
			Adjusted: HR (95% CI)	Unadjusted: HR (95% CI)	Adjusted: HR (95% CI)	Unadjusted: HR (95% CI)
TC	1	With vs. without food intake	0.92 (0.75, 1.13)	0.94 (0.78, 1.15)	0.69 (0.50, 0.95)	0.78 (0.58, 1.06)
	2	With vs. without food intake	0.86 (0.69, 1.08)	0.89 (0.72, 1.11)	0.56 (0.41, 0.77)	0.61 (0.45, 0.83)
	3	With vs. without food intake	0.90 (0.70, 1.15)	0.90 (0.71, 1.14)	0.80 (0.56, 1.13)	0.80 (0.58, 1.12)
FEC	1	With vs. without food intake	0.79 (0.65, 0.96)	0.82 (0.68, 0.98)	0.84 (0.65, 1.08)	0.85 (0.67, 1.09)
	2	With vs. without food intake	0.86 (0.72, 1.03)	0.86 (0.73, 1.02)	0.72 (0.56, 0.92)	0.72 (0.57, 0.91)
	3	With vs. without food intake	0.82 (0.68, 0.99)	0.85 (0.71, 1.01)	0.75 (0.59, 0.97)	0.76 (0.60, 0.97)

NFS and EFS HRs were adjusted, if possible, for predictors age, alcohol per week, smoking, and BMI. Otherwise, the predictors were used as stratification factors. For TC NFS, it was necessary to stratify for age in cycles 1 and for BMI in cycles 2 and 3. For TC EFS, it was necessary to stratify for age in cycles 2 and for BMI in cycles 2. For FEC NFS, it was necessary to stratify for age in cycles 1, 2, and 3. For FEC EFS, it was necessary to stratify for age in cycles 1, 2, and 3, for smoking in cycle 1 and for alcohol in cycles 2 and 3

*HR* hazard ratio, *CI* confidence interval

### NFS and EFS for FEC dependent on pre-cycle meal

For FEC median observation times were 9 (95% CI (6, 72.25)), 11 (95% CI (6, 120)), and 11 (95% CI (6, 120)) hours for NFS and 120 (95% CI (10, 120)), 120 (95% CI (15, 120)), and 120 (95% CI (12, 120)) hours for EFS in cycles 1, 2, and 3, respectively. For FEC, an impact of eating behaviour on NFS was found (*P* = 0.04 in cycle 1, Likelihood ratio test). Furthermore, an impact for EFS was found for eating behaviour (*P* = 0.02 in cycle 2, Likelihood ratio test).

Hazard ratios for NFS and EFS comparing pre-chemotherapy food intake and no pre-chemotherapy food intake for all three cycles are shown in Table 2. For NFS, HR in cycle 1 was 0.79 (95% CI (0.65, 0.96)), and HR in cycle 3 was 0.82 (95% CI (0.68, 0.99)). For EFS, HR in cycle 2 was 0.72 (95% CI (0.56, 0.92)), and HR in cycle 3 was 0.75 (95% CI (0.59, 0.97)). For all other HRs, the 95% CI consisted of values below and above one.

## Discussion

In our large secondary observational analysis of prospectively collected trial data, documented pre-chemotherapy food intake versus no documented pre-chemotherapy food intake on the day of chemotherapy administration significantly improved emesis-free survival in both FEC and TC regimens. Furthermore, an effect on nausea-free survival associated with a pre-chemotherapy meal was observed only in the FEC group.

The number of eligible patients per cycle remained nearly constant. For TC, there were 708, 699, and 696 patients in cycles 1, 2, and 3, respectively, and for FEC, 716,724, and 705 patients in cycles 1, 3, and 3, respectively. In all cycles, both the group with pre-chemotherapy food intake and the group without pre-chemotherapy food intake included more than 220 patients. Thus, results for all cycles are based on sufficiently large subpopulations.

Our data demonstrate differences between patients who consumed a meal right before chemotherapy and those who did not. Differences are observed especially during the first 24 h after chemotherapy infusion (acute phase), as shown in the Kaplan–Meier curves. This is not surprising, as the first emetic or nausea events for both chemotherapy regimens typically occur within 12 h post-chemotherapy [28].

**Fig. 2:**
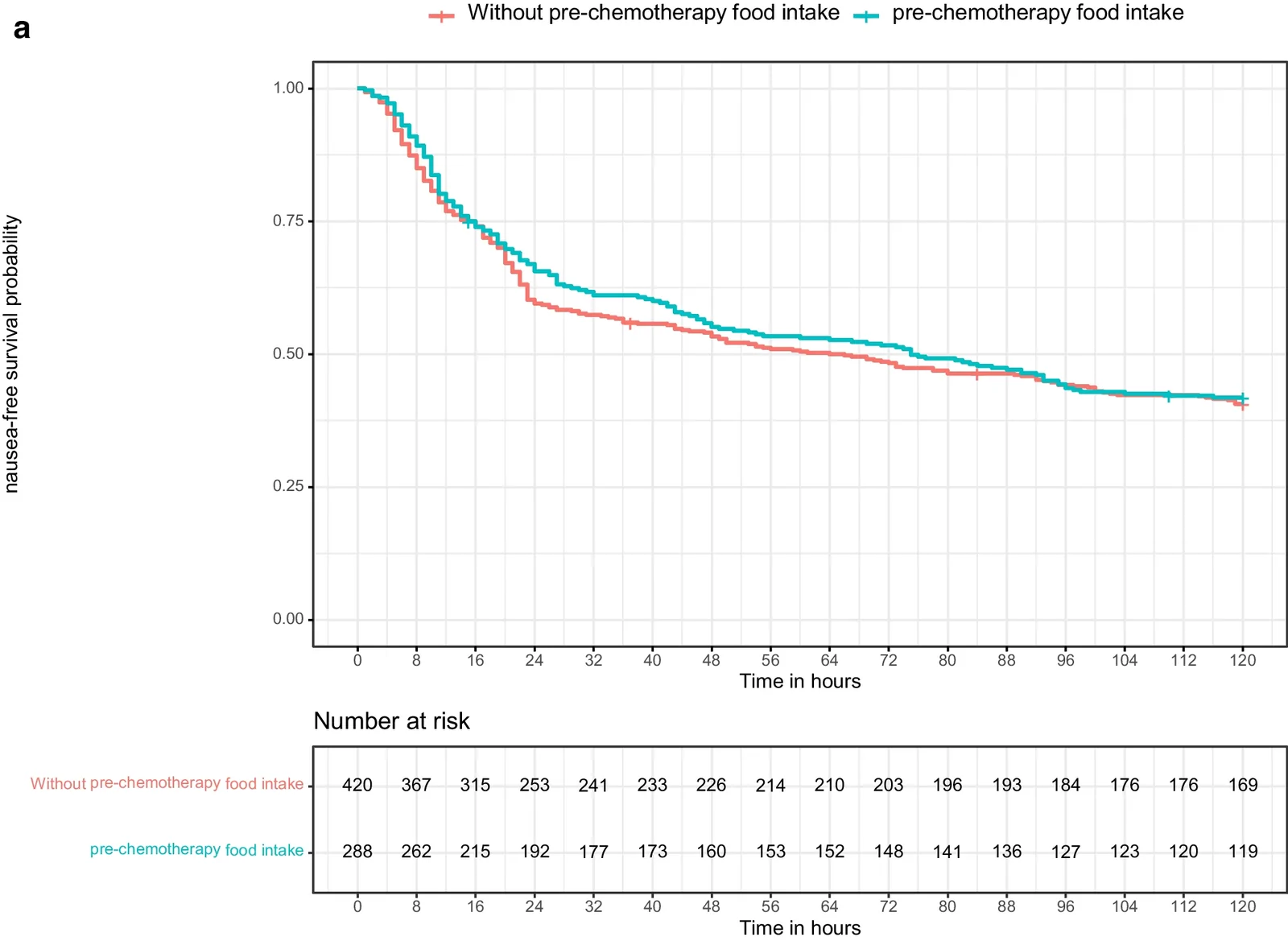

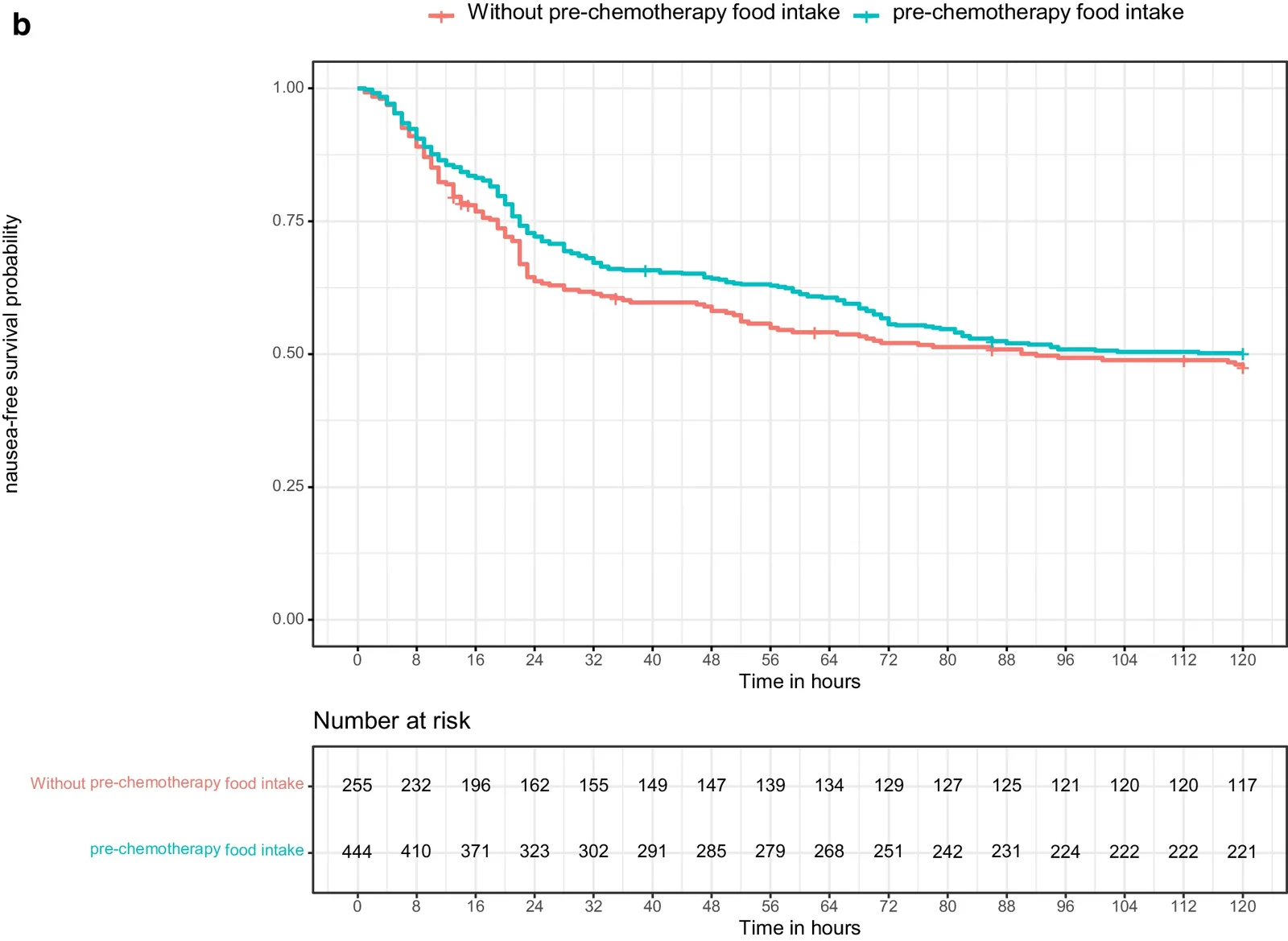

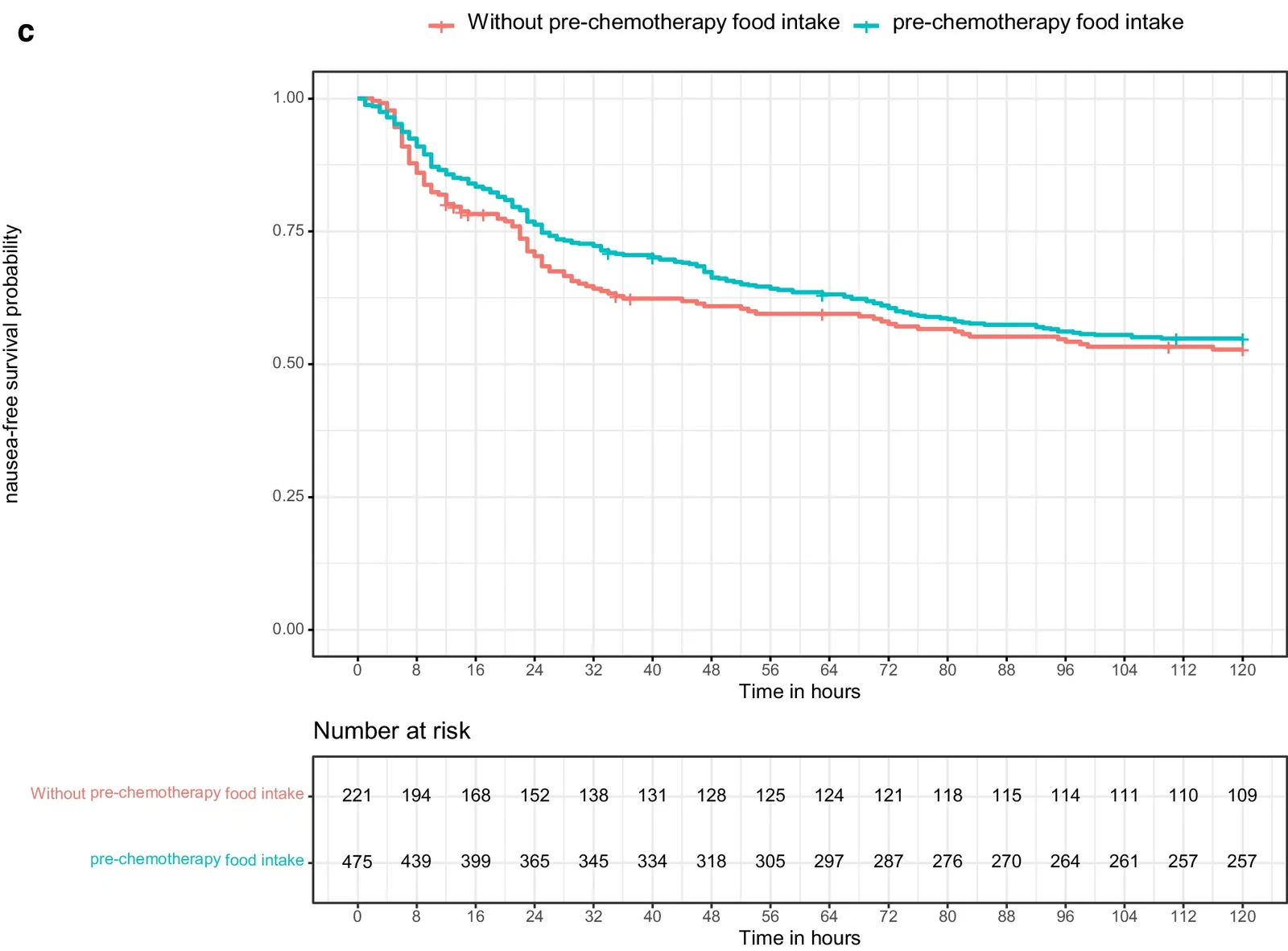

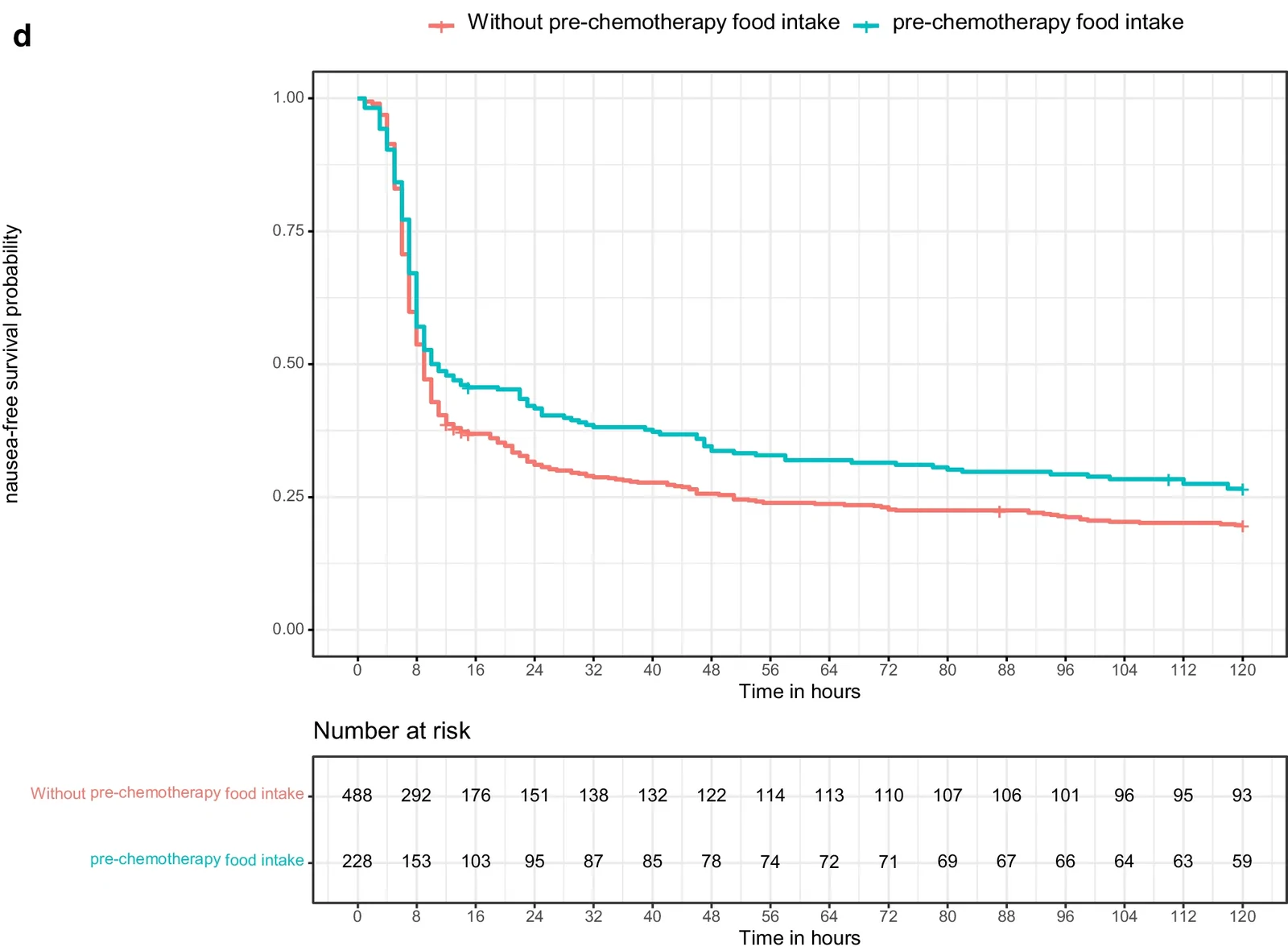

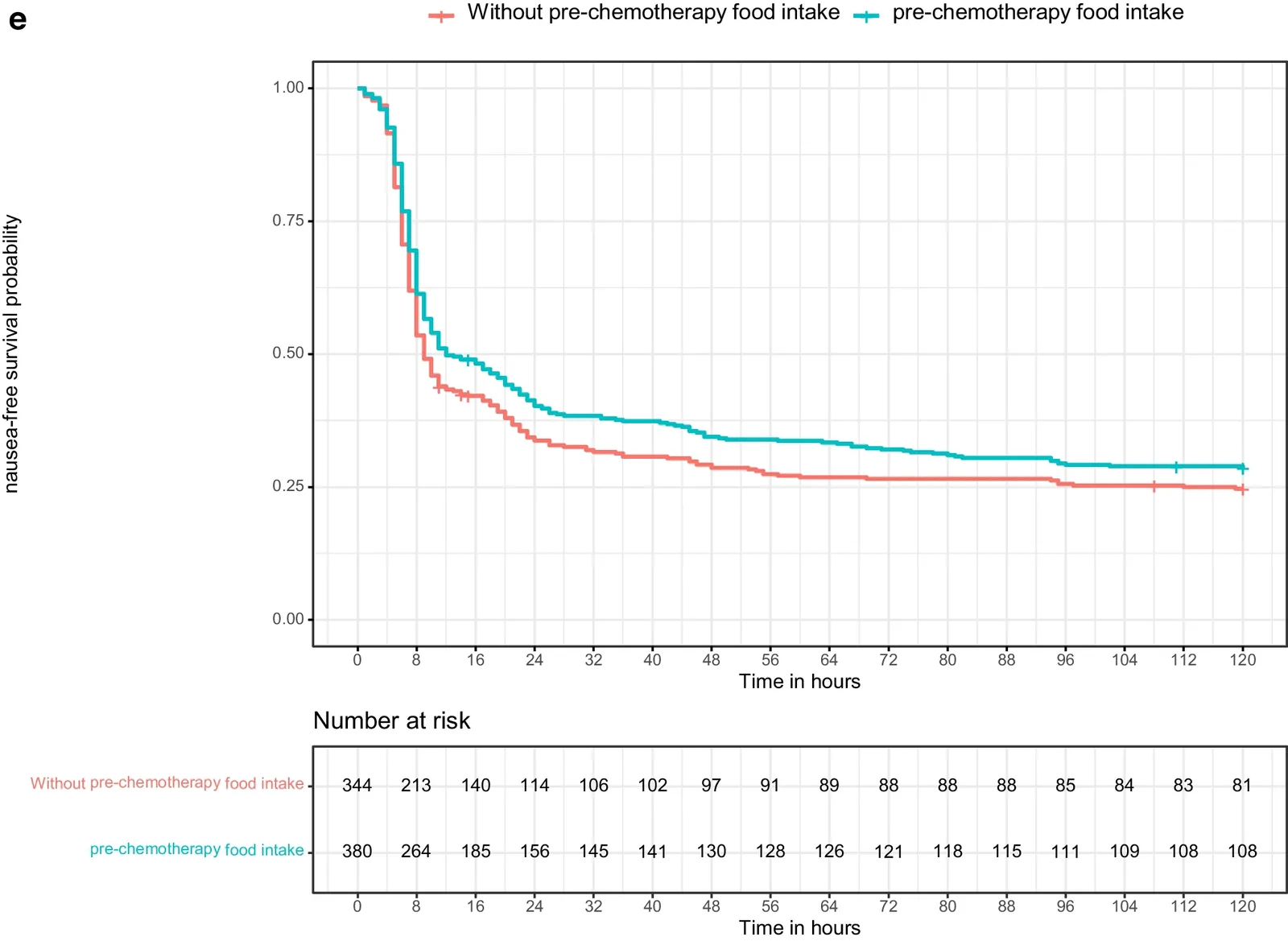

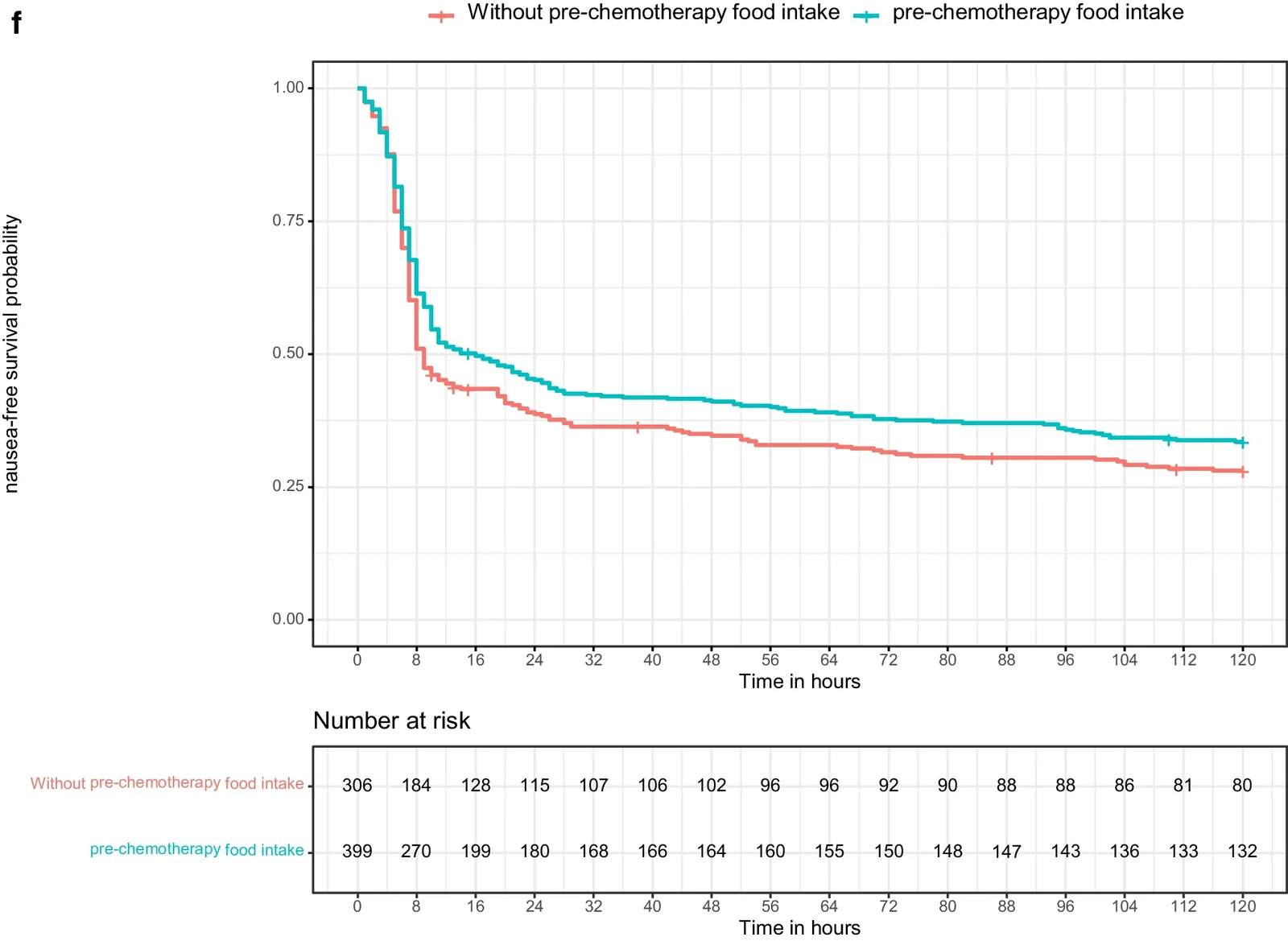
Nausea-free survival for TC and FEC. **a** Nausea-free survival for TC relative to eating behaviour in cycle 1. **b** Nausea-free survival for TC relative to eating behaviour in cycle 2. **c** Nausea-free survival for TC relative to eating behaviour in cycle 3. **d** Nausea-free survival for FEC relative to eating behaviour in cycle 1. **e** Nausea-free survival for FEC relative to eating behaviour in cycle 2. **f** Nausea-free survival for FEC relative to eating behaviour in cycle 3

For TC, no significant effect on NFS was observed. As shown in Fig. 2a–-c, the Kaplan–Meier curves for all three cycles diverge during the first hours after chemotherapy but converge thereafter. Patients who consumed a pre-chemotherapy meal and did not experience nausea on the first day of observation were more likely to develop nausea later on.

No overall effect of pre-chemotherapy eating on CINV was observed. The impact of pre-chemotherapy food intake therefore depends on the endpoint (nausea or emesis) and the chemotherapy regimen (FEC or TC). This should be considered when interpreting the literature on nutritional practices in the context of chemotherapy: conclusions drawn for emesis cannot be directly extrapolated to nausea, nor can findings from one treatment regimen be generalized to another.

Significance only was found within cycles 1 and 2. The observed differences between cycles is more likely explained by a modest effect size that, given the inherent variability of repeated measurements and adjustment for multiple testing, was detectable in some treatment cycles but not others. However, a contribution of anticipatory CINV cannot be entirely excluded.

**Fig. 3:**
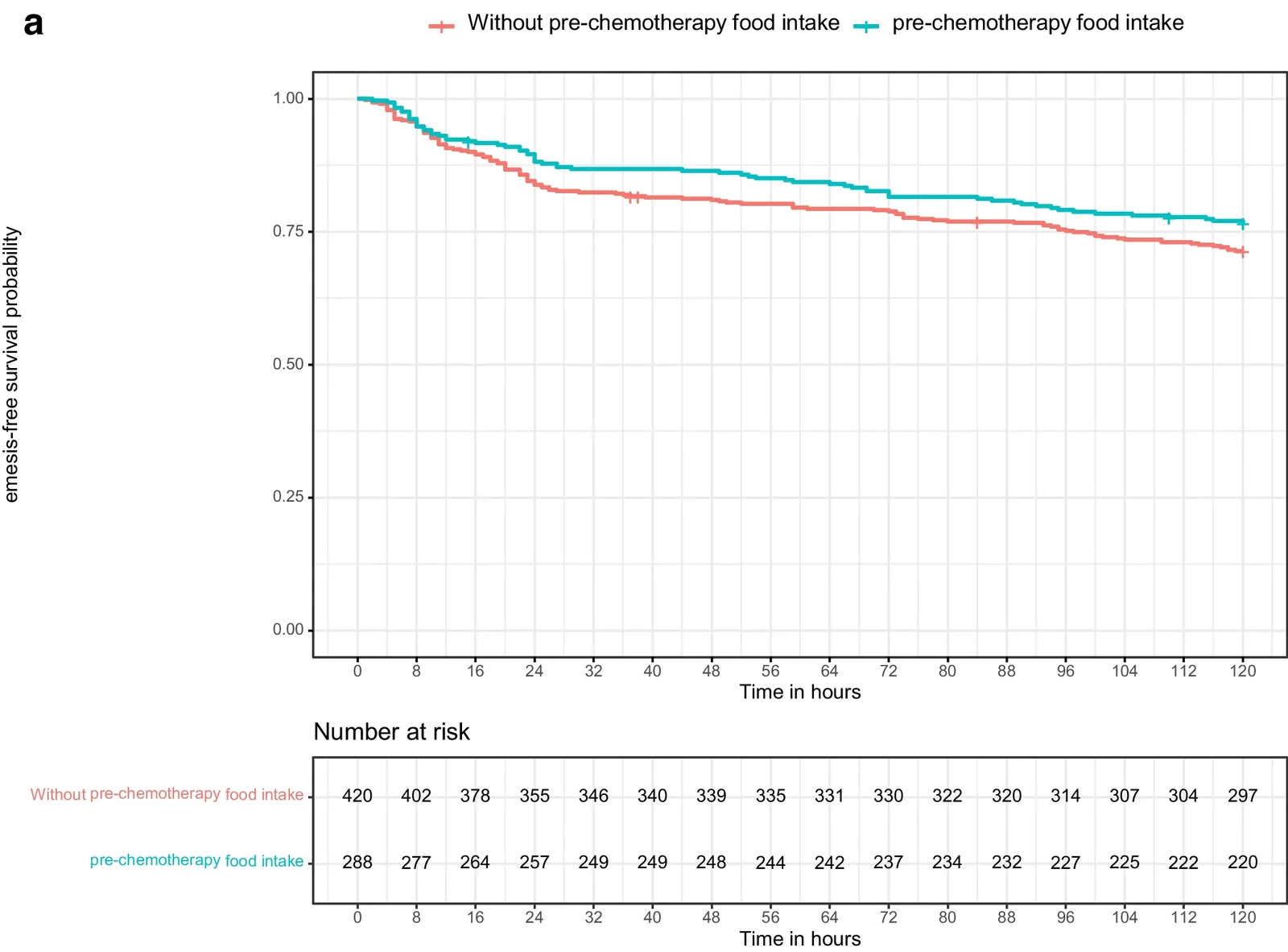

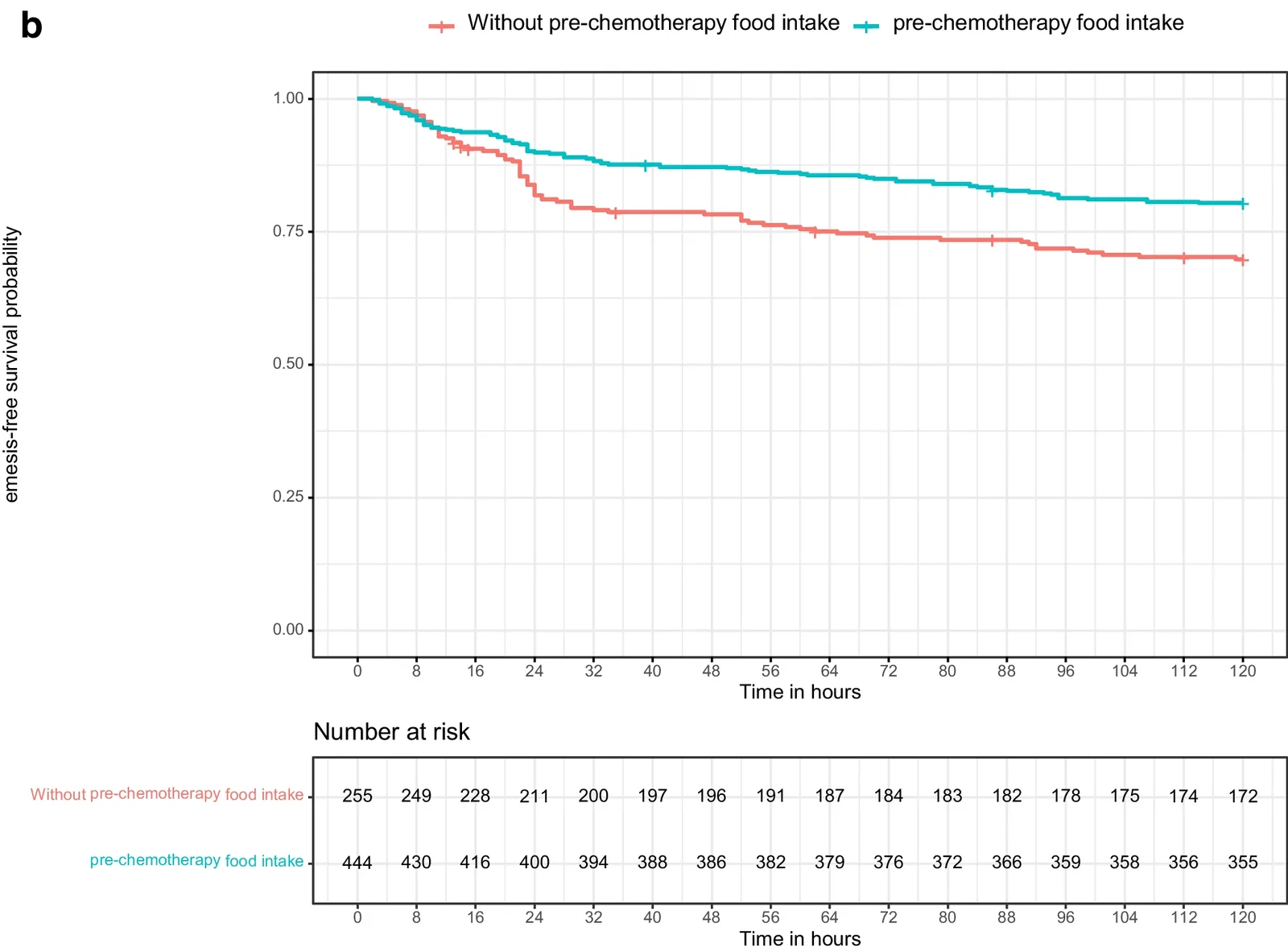

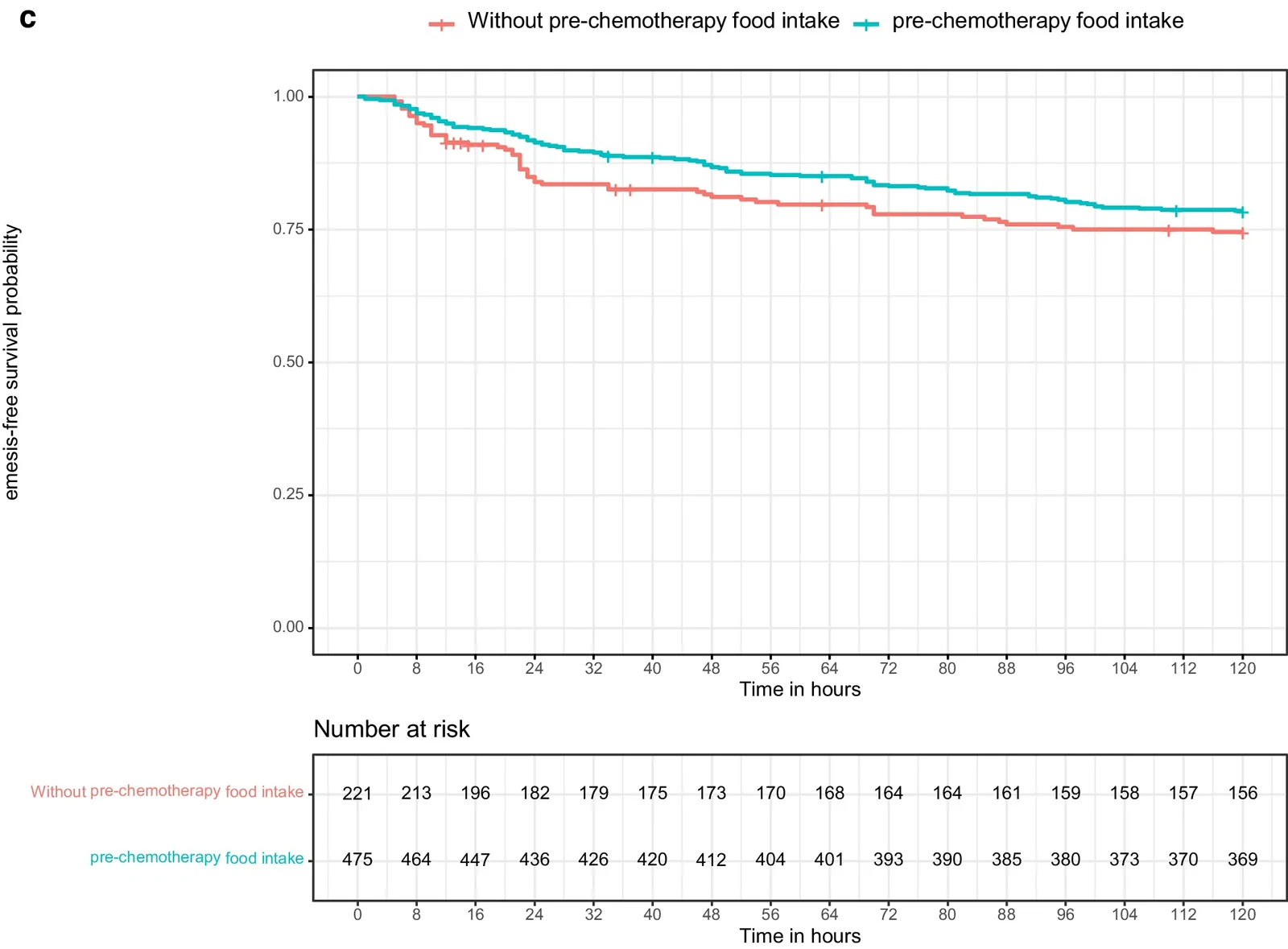

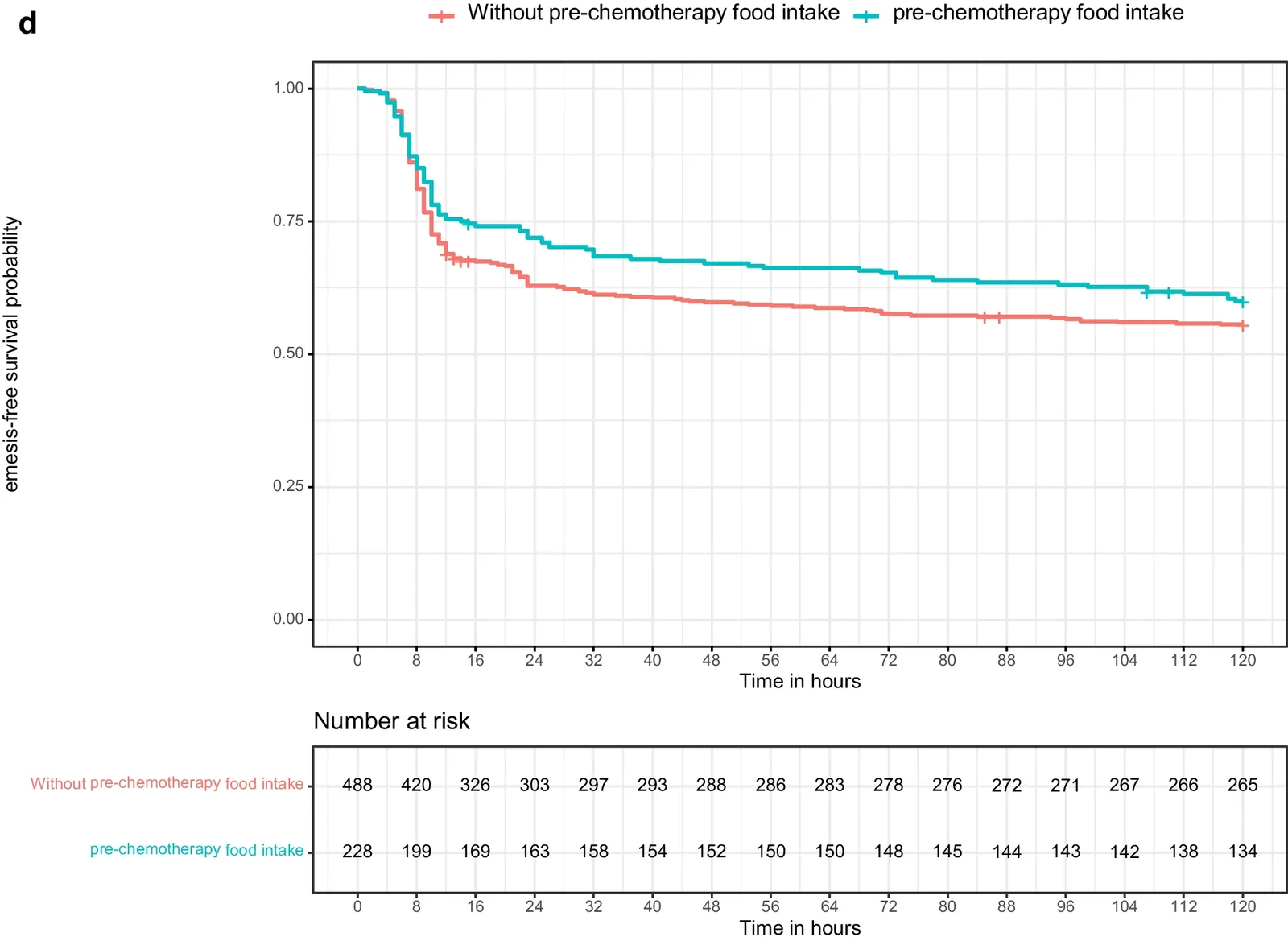

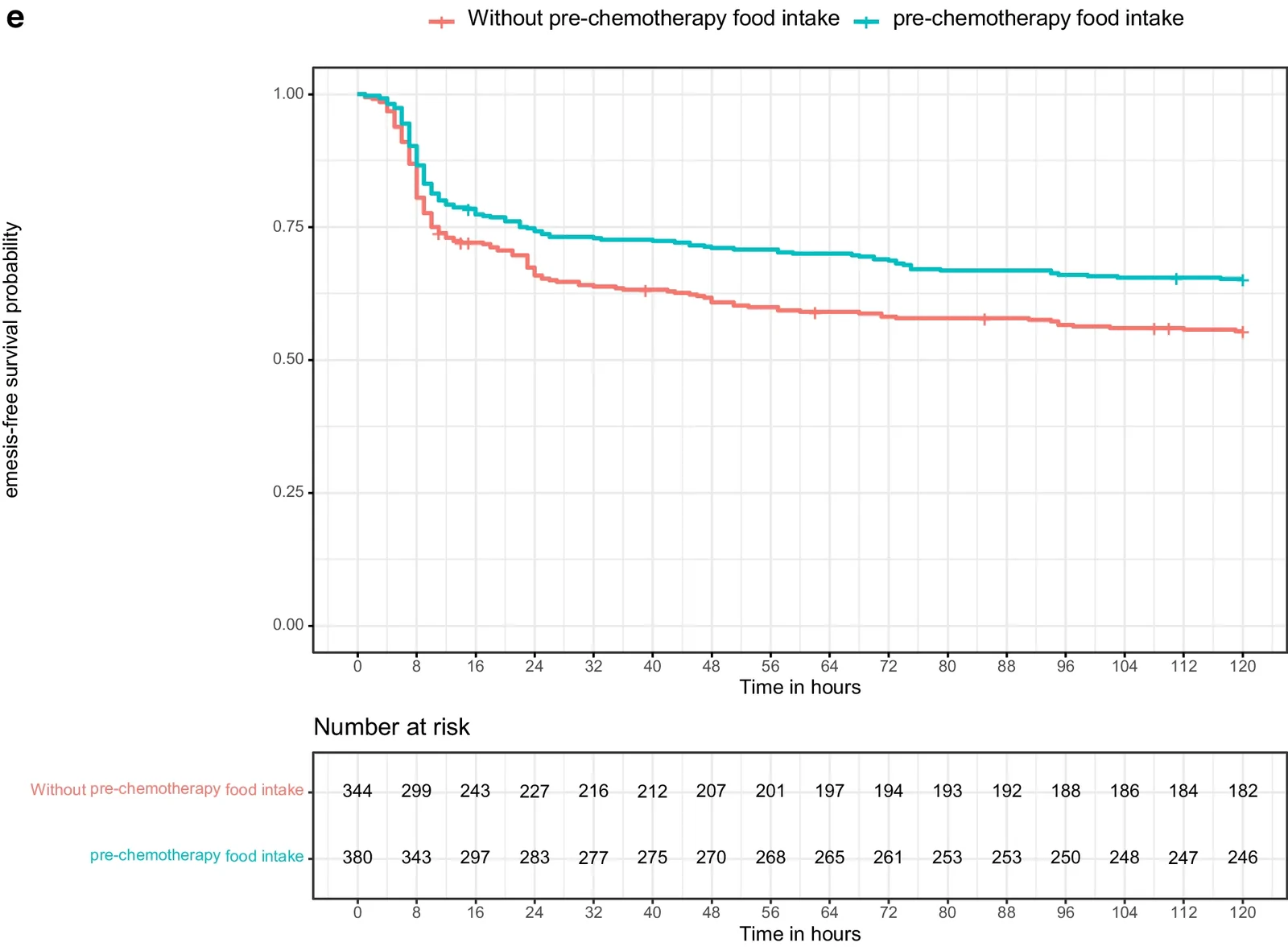

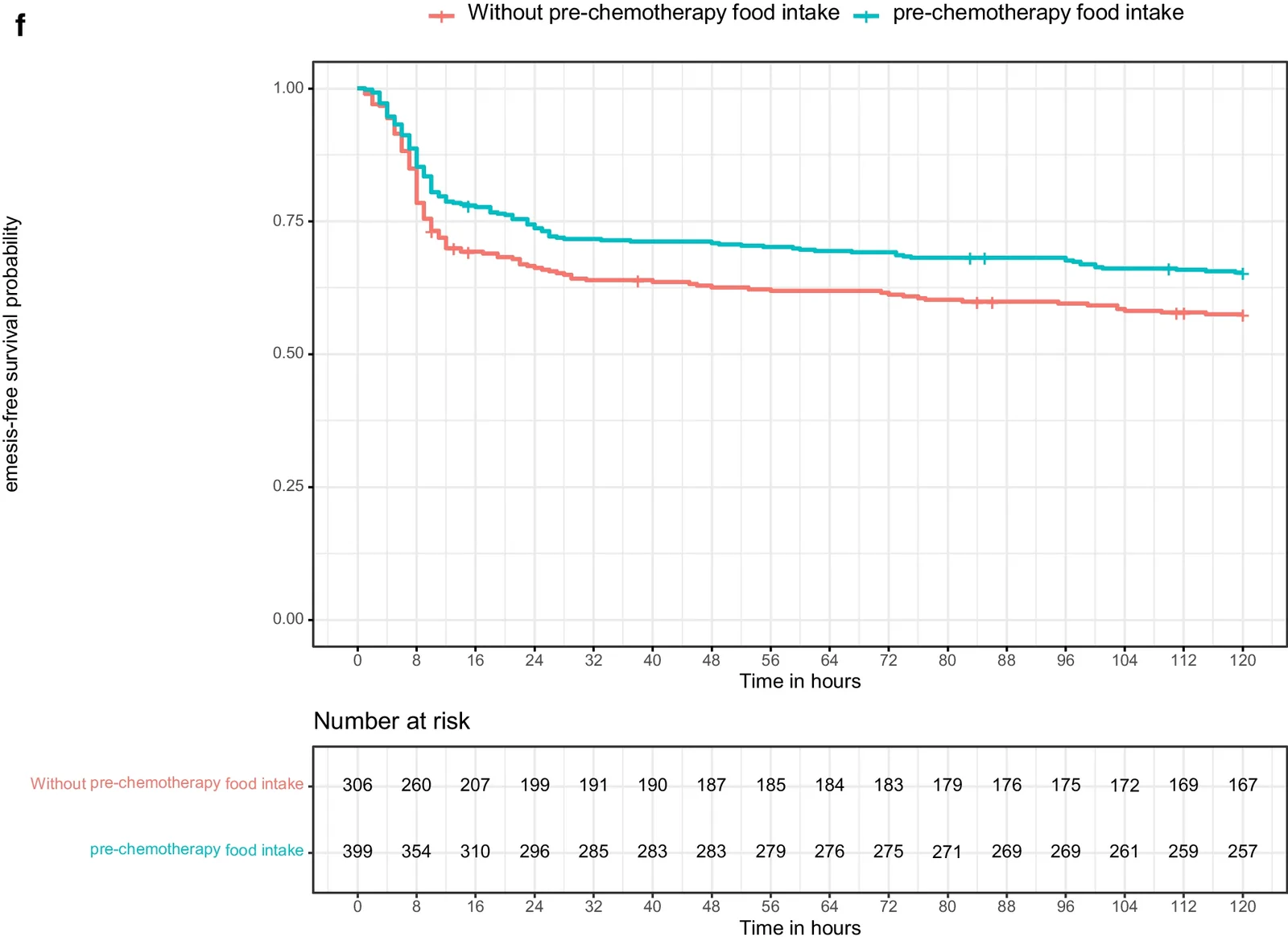
Emesis-free survival for TC and FEC. **a** Emesis-free survival for TC relative to eating behaviour in cycle 1. **b** Emesis-free survival for TC relative to eating behaviour in cycle 2. **c** Emesis-free survival for TC relative to eating behaviour in cycle 3. **d** Emesis-free survival for FEC relative to eating behaviour in cycle 1. **e** Emesis-free survival for FEC relative to eating behaviour in cycle 2. **f** Emesis-free survival for FEC relative to eating behaviour in cycle 3

As shown in the risk tables (Figs. 2 and 3), the majority of patients receiving either TC or FEC did not consume any food prior to chemotherapy in cycle 1. This pattern shifted in cycles 2 and 3. The differences in eating behaviour observed between the first and subsequent treatment cycles may partly reflect increasing familiarity with the treatment process. However, as these behavioural adaptations were not specifically assessed, this explanation remains speculative.

Prophylactic antiemetic protocols differ between regimens, with multi-drug prophylaxis recommended for high (FC) or moderately high (TC) emetic risk. When comparing the observed impact of pre-chemotherapy food intake (with versus without) to the effects of different antiemetic regimens [29–31], the observed effect of food intake supports further investigation of pre-chemotherapy food intake as a potentially modifiable factor influencing CINV outcomes. Although the effect of food intake is likely smaller than that of commonly used antiemetics, it may still be substantial, as observed in our study for TC cycle 2 (HR 0.56 for EFS). Considering the universal necessity of eating and our general result, independent of the type of food, there is no reason not to recommend eating before chemotherapy as complementary action alongside standard antiemetics.

Unfortunately, documentation of antiemetic medication across treatment cycles was incomplete. Table 1 summarizes the overall frequency with which antiemetic medications were documented during the first three chemotherapy cycles, while Supplementary Tables 1 and 2 provide cycle-specific frequencies for the groups with and without food intake before chemotherapy. Only a very small number of antiemetic medications were documented per treatment cycle.

Given that antiemetic prophylaxis was protocol-mandated for all chemotherapy regimens, the available documentation was considered inadequate for meaningful comparative analyses of antiemetic treatment. Consequently, antiemetic medication was not included in the statistical models, and potential interaction effects with eating behaviour could not be assessed reliably. Following potential confounding and interaction effects involving antiemetic medication remain uncertain.

A major strength of this study is the large patient cohort and the comprehensive dataset derived from detailed patient diaries. These enabled high temporal resolution of CINV assessment, including nausea, vomiting, retching, and dietary intake, thus allowing a nuanced analysis of symptom dynamics across both the acute and delayed phases. However, as a secondary observational analysis of prospectively collected trial data, this study was conducted without randomization or intervention. As a result, causal inference regarding the relationship between pre-chemotherapy eating and CINV cannot be drawn, and the possibility of bias due to unmeasured confounders cannot be excluded. Detailed information on meal composition, including caloric and protein content, was not collected. Therefore, a potential influence of specific nutritional characteristics on CINV could not be assessed. A substantial proportion of patients had missing diary data, with considerable variation in data availability between centres. Although this suggests a relevant centre-specific effect, we cannot exclude that symptom severity or poor general condition contributed to non-completion, potentially introducing selection bias. However, this is the largest study to date investigating the impact of pre-chemotherapy food intake on chemotherapy-induced nausea and vomiting, providing an important first contribution to understanding this relationship and a valuable basis for future studies.

## Conclusion

In conclusion, this analysis represents the largest analysis examining CINV-parameters and outcomes in patients with early breast cancer receiving chemotherapy. Pre-chemotherapy food intake versus no pre-chemotherapy food intake was associated with prolonged emesis-free survival for both TC and FEC regimes, and with extended nausea-free survival in the FEC group. Differences were most prominent during the first 24 h after chemotherapy.

Compared to prior smaller studies, our data support a significant effect of pre-chemotherapy food intake. Given the secondary observational nature of this analysis based on prospectively collected trial data, prospective trials focusing on patient education and pre-treatment nutrition are warranted. Future research should also investigate the potential role of additional supportive strategies in patients receiving novel antibody-drug conjugates (ADCs). While these agents have demonstrated improved clinical efficacy in several treatment settings, they are associated with distinct toxicity profiles, including nausea and vomiting, that may require tailored supportive care approaches [32]. Prolonged CINV remains a clinically relevant concern with some ADCs [33, 34], while evidence regarding optimal pharmacological antiemetic strategies is less established than for conventional chemotherapy. This highlights the need to investigate additional supportive approaches in this setting. Given its simplicity and feasibility, pre-treatment food intake may represent one such approach worthy of prospective investigation.

## Supplementary Information

Below is the link to the electronic supplementary material.

ESM 1 (DOCX 34.6 KB)

ESM 2 (PDF 155 KB)

## Data Availability

The datasets used and/or analyzed during the current study can be requested in context of a research project regarding chemotherapy induced nausea and vomiting. Proposals are evaluated by the SUCCESS C scientific board. In case of approval, data is available from the corresponding author.

## References

[1] Yeo W et al (2021) Quality of life associated with nausea and vomiting from anthracycline-based chemotherapy: a pooled data analysis from three prospective trials. Oncologist 26(12):e2288–e2296 10.1002/onco.13978 PMC8648999 34516038

[2] Sommariva S, Pongiglione B, Tarricone R (2016) Impact of chemotherapy-induced nausea and vomiting on health-related quality of life and resource utilization: a systematic review. Crit Rev Oncol Hematol 99:13–36 10.1016/j.critrevonc.2015.12.001 26697988

[3] Fernández-Ortega P et al (2012) Chemotherapy-induced nausea and vomiting in clinical practice: impact on patients’ quality of life. Support Care Cancer 20(12):3141–3148 10.1007/s00520-012-1448-1 22460057

[4] Gupta K, Walton R, Kataria SP (2021) Chemotherapy-induced nausea and vomiting: pathogenesis, recommendations, and new trends. Cancer Treat Res Commun 26:100278 10.1016/j.ctarc.2020.100278 33360668

[5] Rugo HS et al (2022) Optimizing treatment management of trastuzumab deruxtecan in clinical practice of breast cancer. ESMO Open 7:100553 10.1016/j.esmoop.2022.100553 PMC9375150 35964548

[6] Navari RM, Aapro M (2016) Antiemetic prophylaxis for chemotherapy-induced nausea and vomiting. N Engl J Med 374(14):1356–1367 10.1056/NEJMra1515442 27050207

[7] Onkologie L (2025) *Leitlinienprogramm Onkologie: Supportive Therapie*. 23 August 2025]; Available from: https://www.leitlinienprogramm-onkologie.de/leitlinien/supportive-therapie

[8] Mosa ASM et al (2020) Patient-related risk factors for chemotherapy-induced nausea and vomiting: a systematic review. Front Pharmacol 11:329 10.3389/fphar.2020.00329 PMC7138899 32296333

[9] Booth CM et al (2007) Chemotherapy-induced nausea and vomiting in breast cancer patients: a prospective observational study. J Support Oncol 5(8):374–380 17944146

[10] Gala D et al (2022) Dietary strategies for chemotherapy-induced nausea and vomiting: a systematic review. Clin Nutr 41(10):2147–2155 10.1016/j.clnu.2022.08.003 36067586

[11] (DGEM), D.G.f.E. *S3-Leitlinie Klinische Ernährung in der Onkologie*. 2015 [cited 2025 2 August 2025]; Available from: https://www.dgem.de/sites/default/files/PDFs/Leitlinien/S3-Leitlinien/073-006l_S3_Klin_Ern%C3%A4hrung_in_der_Onkologie_2015-10.pdf

[12] Abdollahi R et al (2019) The effect of dietary intervention along with nutritional education on reducing the gastrointestinal side effects caused by chemotherapy among women with breast cancer. Nutr Cancer 71(6):922–930 10.1080/01635581.2019.1590608 30945949

[13] Souza APS, Silva LCD, Fayh APT (2021) Nutritional intervention contributes to the improvement of symptoms related to quality of life in breast cancer patients undergoing neoadjuvant chemotherapy: a randomized clinical trial. Nutrients 13(2):589 10.3390/nu13020589 PMC7916763 33579050

[14] Lou Y et al (2014) Self-management of chemotherapy-related nausea and vomiting: a cross-sectional survey of Chinese cancer patients. Cancer Nurs 37(2):126–138 10.1097/NCC.0b013e318291b6f5 23666267

[15] Hesketh PJ et al (2010) Evaluation of risk factors predictive of nausea and vomiting with current standard-of-care antiemetic treatment: analysis of two phase III trials of aprepitant in patients receiving cisplatin-based chemotherapy. Support Care Cancer 18(9):1171–1177 10.1007/s00520-009-0737-9 19756774

[16] Marx W et al (2016) Chemotherapy-induced nausea and vomiting: a narrative review to inform dietetics practice. J Acad Nutr Diet 116(5):819–827 10.1016/j.jand.2015.10.020 26686816

[17] Jednak MA et al (1999) Protein meals reduce nausea and gastric slow wave dysrhythmic activity in first trimester pregnancy. Am J Physiol 277(4):G855–G861 10.1152/ajpgi.1999.277.4.G855 10516152

[18] Levine ME et al (2008) Protein and ginger for the treatment of chemotherapy-induced delayed nausea. J Altern Complement Med 14(5):545–551 10.1089/acm.2007.0817 18537470

[19] Levine ME et al (2004) Protein-predominant meals inhibit the development of gastric tachyarrhythmia, nausea and the symptoms of motion sickness. Aliment Pharmacol Ther 19(5):583–590 10.1111/j.1365-2036.2004.01885.x 14987327

[20] Ligibel JA et al (2022) Exercise, diet, and weight management during cancer treatment: ASCO Guideline. J Clin Oncol 40(22):2491–2507 10.1200/JCO.22.00687 35576506

[21] Herrstedt J et al (2023) 2023 MASCC and ESMO guideline update for the prevention of chemotherapy- and radiotherapy-induced nausea and vomiting. ESMO Open 9(2):102195 10.1016/j.esmoop.2023.102195 PMC10937211 38458657

[22] de Gregorio A et al (2022) The impact of anthracyclines in intermediate and high-risk HER2-negative early breast cancer—a pooled analysis of the randomised clinical trials PlanB and SUCCESS C. Br J Cancer 126:1715–1724 10.1038/s41416-021-01690-6 PMC9174181 35194193

[23] Rack B et al (2010) The German SUCCESS C study - the first European lifestyle study on breast cancer. Breast Care (Basel) 5(6):395–400 10.1159/000322677 PMC3076352 21494405

[24] Lux MP, Maass N, Schütz F, Schwidde I, Fasching PA, Fehm T, Janni W, Kümmel S, Kolberg H-C, Lüftner D (2013) Breast cancer 2013 – interpretation of new and known data. Geburtshilfe Frauenheilkd 73(06):584–598

[25] Schane R, Ling P, Glantz S (2010) Health effects of light and intermittent smoking: a review. Circulation 21(13):1518–1522. 10.1161/CIRCULATIONAHA.109.904235 PMC2865193 20368531

[26] Grambsch PM, Therneau TM (1994) Proportional hazards tests and diagnostics based on weighted residuals. Biometrika 81(3):515–526

[27] Therneau T, Crowson C, Atkinson E (2024) *Using time dependent covariates and time dependent coefficients in the cox model. Vignette of R package survival*. [cited 2025 03.08.2025]; Available from: https://cran.r-project.org/web/packages/survival/vignettes/timedep.pdf

[28] Hörner M et al (2026) Chemotherapy-induced nausea and vomiting in early breast cancer patients receiving adjuvant chemotherapy with fluorouracil, epirubicin, cyclophosphamide followed by docetaxel versus an anthracycline-free regimen with docetaxel, cyclophosphamide—results from a randomized clinical trial. Int J Cancer 159(8):1990–2003. 10.1002/ijc.70528 PMC13495824 42056728

[29] Filetti M et al (2023) Efficacy and safety of antiemetic regimens for highly emetogenic chemotherapy-induced nausea and vomiting: a systematic review and network meta-analysis. Cancer Treat Rev 115:102512 10.1016/j.ctrv.2023.102512 36774658

[30] Llombart-Cussac A et al (2009) Incidence of chemotherapy-induced nausea and vomiting associated with docetaxel and cyclophosphamide in early breast cancer patients and aprepitant efficacy as salvage therapy. Results from the Spanish Breast Cancer Group/2009-02 study. Eur J Cancer 58:122–129 10.1016/j.ejca.2016.01.015 26994459

[31] Kawazoe H et al (2018) Olanzapine plus aprepitant, palonosetron, and dexamethasone for nausea and vomiting in patients with breast cancer receiving anthracycline: a retrospective study. Sci Rep 8(1):16232 10.1038/s41598-018-34618-x PMC6214966 30389996

[32] Würstlein R et al (2024) Update Mammakarzinom 2024 Teil 1 – Expertenmeinungen zu Brustkrebs in fortgeschrittenen Krankheitsstadien. Geburtshilfe Frauenheilkd 84(06):487

[33] Perachino M et al (2025) Adverse events and impact on quality of life of antibody-drug conjugates in the treatment of metastatic breast cancer: a systematic review and meta-analysis. Eur J Clin Invest 55(6):e70001 10.1111/eci.70001 PMC12066883 39943891

[34] Park YH et al (2025) Nausea and vomiting in an evolving anticancer treatment landscape: long-delayed and emetogenic antibody-drug conjugates. Future Oncol 21(10):1261–1272 10.1080/14796694.2025.2479417 PMC11988240 40105595

