# Supplementary material for: Impact of pre-chemotherapy food intake on chemotherapy-induced nausea and vomiting in patients with early breast cancer: a subanalysis of the randomized SUCCESS C trial
Source: Support Care Cancer. 2026 Nov 13;34(12):1223. doi: 10.1007/s00520-026-11437-9 (PMC13768299; doi:10.1007/s00520-026-11437-9)
Supplement: Supplementary file 1 [file 520_2026_11437_MOESM2_ESM.pdf]

8.6 Emesetagebuch

Sehr geehrte Patientin,

Es freut uns, dass Sie an der Success- C Studie teilnehmen.
Dieses Tagebuch dient dazu, dass Sie genaue Angaben zu Übelkeit und Episoden von Emesis (d.h. Erbrechen oder Brechreiz) machen.
Ihr Arzt hat Ihnen bereits gezeigt, wie Sie das Tagebuch benutzen sollten, hier erklären wir Ihnen nochmals kurz die wichtigsten Dinge, bei Unklarheiten wenden Sie sich gerne jederzeit an Ihren betreuenden Studienarzt.

Definitionen

Emesis:
Brechreiz und / oder Erbrechen.
Erbrechen:
Ausstoßen von Mageninhalt durch Mund oder Nase.
Brechreiz:
Versuch, sich zu erbrechen, der nicht zum Ausstoßen von Mageninhalt führt.

Anleitung zum Ausfüllen des Tagebuches

Wann mache ich die Tagebucheintragungen ?

Den ersten Eintrag nehmen Sie bitte an dem Tag, an dem Sie Ihre Chemotherapie bekommen, vor.
Die nächsten Einträge machen Sie bitte ab dem ersten Tag nach der Chemotherapie bis zum 5. Tag nach der Chemotherapie. Wenn Sie z.B. montags ihre erste Chemotherapie erhalten, nehmen Sie die Tagebucheinträge von montags bis samstags vor.

Was muss ich bei den Eintragungen beachten ?

Das Tagebuch besteht hauptsächlich aus einer Tabelle, in dieser sind oben die Uhrzeiten nach der 24-Stunden-Uhr notiert. Bitte machen Sie jeweils einen senkrechten Strich, wenn Sie z.B. erbrochen haben oder etwas gegessen und getrunken haben. Haben Sie innerhalb einer Stunde mehrmals erbrochen, z.B. dreimal, machen Sie entsprechend bitte 3 Striche.
Wenn Sie die täglichen Beurteilungen ausfüllen, gibt es eine Besonderheit und zwar bei der Dokumentation der Übelkeit: Bitte schreiben Sie den Schweregrad der Übelkeit (Einteilung steht über der Tabelle) anstelle davon, dass Sie nur einen Strich machen.
Haben Sie zusätzlich Medikamente gegen Übelkeit oder Erbrechen genommen, nummerieren Sie bitte die zusätzlich genommenen Medikamente durch, schreiben Sie in der Tabelle die Nummer und in der Aufstellung unter der Tabelle unter der entsprechenden Nummer, von welchem Medikament sie wie viel genommen haben.
Für uns ist es eine große Hilfe, wenn Sie sauber und gut lesbar mit einem schwarzen Kugelschreiber schreiben.
In dem Fall, dass Sie etwas falsch eingetragen haben, streichen Sie bitte diesen Eintrag mit einer einfachen Linie durch und schreiben den korrekten Eintrag daneben. Bitte zeichnen Sie die Korrektur mit Ihren Initialen und dem Datum ab.

Beispiel:

Eine Patientin erhält am Montag, den 02.03.2009 um 9.30 Uhr morgens die Chemotherapie.

Sie isst und trinkt um 12 und um 16 Uhr.

Um 17 Uhr hat sie starkes Übelkeitsgefühl.

Zwischen 18 und 19 Uhr erbricht sie zweimal.

Um 20 Uhr nimmt sie 20 Tropfen MCP.

Tag der Chemotherapie, Datum: 02.03.2009__

Uhrzeit der Chemotherapie: 9:30 Uhr

Bitte die Schweregrade der Übelkeit folgendermaßen angeben:

Grad 1 (1): leichte Übelkeit (Übelkeit, Magenverstimmung, die beherrschbar ist und die Alltagsaktivitäten, wenn überhaupt, nur minimal beeinflusst)

Grad 2 (2): mittelschwere Übelkeit (stärkere Übelkeit, die manchmal mit dem Gefühl verbunden ist, erbrechen zu müssen, jedoch ohne tatsächliches Erbrechen, Alltagsaktivitäten wie Arbeiten, Essen, Trinken, Zubereiten einer Mahlzeit,… sind erschwert

Grad 3 (3): starke Übelkeit (sehr starke Übelkeit oder Gefühl, dass man sich fast dauernd übergeben muss, wobei man die Alltagsaktivitäten nicht ausführen kann)

	0 Uhr	1	2	3	4	5	6	7	8	9	10	11	12	13	14	15	16	17	18	19	20	21	22	23
Übelkeit																		3						

(bitte Schweregrad angeben)																								
Brechreiz																								
Erbrechen																		11						
Einnahme von anderen Medikamenten																				1				
Gegessen												1				1								
Getrunken												1				1								

Falls Sie zusätzlich zur Studienmedikation Medikamente gegen Übelkeit und Erbrechen/Brechreiz genommen haben geben Sie bitte hier die Uhrzeit der Einnahme, Namen des Präparates, Dosierung, Anzahl der Einnahmen und die Darreichungsform des Medikaments an.
Bitte tragen Sie je Zeile nur ein Medikament ein. Tragen Sie dann in die oben stehende Tabelle bei der entsprechenden Uhrzeit die Nummer ein.

Nummer	Uhrzeit	Name des Medikaments	Dosierung	Anzahl der Einnahme	Darreichungsform
1	20 Uhr	MCP	20	1	☐ Tablette<br>☐ Zäpfchen<br>X Tropfen
2					☐ Tablette<br>☐ Zäpfchen<br>☐ Tropfen
3					☐ Tablette<br>☐ Zäpfchen<br>☐ Tropfen
4					☐ Tablette<br>☐ Zäpfchen<br>☐ Tropfen
5					☐ Tablette<br>☐ Zäpfchen<br>☐ Tropfen
6					☐ Tablette<br>☐ Zäpfchen<br>☐ Tropfen
7					☐ Tablette<br>☐ Zäpfchen<br>☐ Tropfen

8					☐ Tablette<br>☐ Zäpfchen<br>☐ Tropfen

Am Ende des Tages: Bitte beurteilen Sie den schlimmsten Grad der Übelkeit, der bei Ihnen seit der Chemotherapie aufgetreten ist, indem Sie einen senkrechten Strich (|) auf der nachstehenden Linie eintragen.

Keine Übelkeit ◆————————————◆ Schlimmste Form der Übelkeit

Bevor Sie mit der Chemotherapie beginnen, würden wir Sie um ein paar zusätzliche Informationen bitten, die uns helfen, Ihre Brustkrebserkrankung oder Ihre Veranlagung für Übelkeit oder Erbrechen besser zu verstehen.
Dafür möchten wir Ihnen zunächst ein paar allgemeine Fragen stellen.

Frage 1 Wie oft pro Tag essen Sie Obst oder Gemüse in einzelnen Portionen?	☐ Ich esse Obst und/oder Gemüse eher unregelmäßig<br>☐ Ich esse Obst und/oder Gemüse ca. 1mal pro Tag<br>☐ Ich esse Obst und/oder Gemüse ca. 2 - 3mal pro Tag<br>☐ Ich esse Obst und/oder Gemüse ca. 4 - 5mal pro Tag
Frage 2 Achten Sie bewusst auf eine fettarme Ernährung?	☐ Nein ☐ Ja, ich ernähre mich vorwiegend fettarm<br>☐ Kann ich nicht beurteilen
++<br>Frage 3 Wie oft in der Woche trinken Sie ein alkoholisches Getränk?	☐ Nie<br>☐ 1-2mal pro Woche<br>☐ 3-6mal pro Woche<br>☐ Eher Jeden Tag

Frage 4: Wie oft waren Sie schwanger?	_____________mal schwanger
Frage 5: Wie viele Kinder haben Sie geboren?	_____________(Anzahl der Kinder)
Frage 6: Wie alt waren Sie bei der ersten Geburt?	_____________(Alter bei erster Geburt)
Frage 7: In welchem Alter hatten Sie ihre erste Periodenblutung?	_____________(Alter bei erster Menstruation)
Frage 8: Haben Sie Hormone nach den Wechseljahren als Pille, Pflaster, Zäpfchen oder Creme eingenommen?	☐ Nein, nie<br>☐ Ja, bis zum Zeitpunkt meiner Brustkrebserkrankung<br>☐ Ja, früher, aber nicht mehr zum Zeitpunkt meiner Brustkrebserkrankung
Frage 9: Hatte Ihre Mutter Brustkrebs?	☐ Nein ☐ Ja
Frage 10: Wie viele Schwestern haben Sie?	_____________Schwestern
,<br>Frage 11: Wie viele Ihrer Schwestern haben/hatten eine Brustkrebserkrankung?	____________Schwestern hatten Brustkrebs<br>(Bitte „0“ eintragen, wenn keine)
1<br>Frage 12: Wie viele Tanten mütterlicherseits haben Sie?	____________Tanten mütterlicherseits<br>(Bitte „0“ eintragen, wenn keine)

Frage 13: Wie viele Tanten mütterlicherseits haben eine Brustkrebserkrankung?	____________dieser Tanten hatten Brustkrebs (Bitte „0“ eintragen, wenn keine)
Frage 14: Wie viele Tanten väterlicherseits haben Sie?	___________Tanten väterlicherseits (Bitte „0“ eintragen, wenn keine)
Frage 15: Wie viele Tanten väterlicherseits haben/hatten eine Brustkrebserkrankung?	___________dieser Tanten hatten Brustkrebs (Bitte „0“ eintragen, wenn keine)
Frage 16: Wie viele ihrer Großmütter haben/hatten eine Bruskrebserkrankung?	___________Großmütter hatten Brustkrebs (Bitte „0“ eintragen, wenn keine)

Nun möchten wir Ihnen noch 2 Fragen zum Vorkommen von Eierstockkrebs in der Familie stellen.

Frage 17: Hatte Ihre Mutter Eierstockkrebs?	☐ Nein	☐ Ja
Frage 18: Wie viele Ihrer Schwestern haben Eierstockkrebs?	__________Schwestern hatten Eierstockkrebs (Bitte „0“ eintragen, wenn keine)	

SUCCESS C

Tagebuch für den Tag der Chemotherapie

Datum: _______________

Uhrzeit der Chemotherapie: _______________

Bitte die Schweregrade der Übelkeit folgendermaßen angeben:

Grad 1 (1): leichte Übelkeit (Übelkeit, Magenverstimmung, die beherrschbar ist und die Alltagsaktivitäten, wenn überhaupt, nur minimal beeinflusst)

Grad 2 (2): mittelschwere Übelkeit (stärkere Übelkeit, die manchmal mit dem Gefühl verbunden ist, erbrechen zu müssen, jedoch ohne tatsächliches Erbrechen, Alltagsaktivitäten wie Arbeiten, Essen, Trinken, Zubereiten einer Mahlzeit,… sind erschwert

Grad 3 (3): starke Übelkeit (sehr starke Übelkeit oder Gefühl, dass man sich fast dauernd übergeben muss, wobei man die Alltagsaktivitäten nicht ausführen kann)

	0 Uhr	1	2	3	4	5	6	7	8	9	10	11	12	13	14	15	16	17	18	19	20	21	22	23
Übelkeit (bitte Schweregrad angeben)																								
Brechreiz																								
Erbrechen																								
Einnahme von anderen Medikamenten																								
Gegessen																								
Getrunken																								

Falls Sie zusätzlich zur Studienmedikation Medikamente gegen Übelkeit und Erbrechen/Brechreiz genommen haben geben Sie bitte hier die Uhrzeit der Einnahme, Namen des Präparates, Dosierung, Anzahl der Einnahmen und die Darreichungsform des Medikaments an.
Bitte tragen Sie je Zeile nur ein Medikament ein. Tragen Sie dann in die oben stehende Tabelle bei der entsprechenden Uhrzeit die Nummer ein.

Nummer	Uhrzeit	Name des Medikaments	Dosierung	Anzahl der Einnahme	Darreichungsform
1					☐ Tablette<br>☐ Zäpfchen<br>☐ Tropfen
2					☐ Tablette<br>☐ Zäpfchen<br>☐ Tropfen
3					☐ Tablette<br>☐ Zäpfchen<br>☐ Tropfen
4					☐ Tablette<br>☐ Zäpfchen<br>☐ Tropfen
5					☐ Tablette<br>☐ Zäpfchen<br>☐ Tropfen
6					☐ Tablette<br>☐ Zäpfchen<br>☐ Tropfen
7					☐ Tablette<br>☐ Zäpfchen<br>☐ Tropfen
8					☐ Tablette

					☐ Zäpfchen<br>☐ Tropfen

Am Ende des Tages: Bitte beurteilen Sie den schlimmsten Grad der Übelkeit, der bei Ihnen seit der Chemotherapie aufgetreten ist, indem Sie einen senkrechten Strich (|) auf der nachstehenden Linie eintragen.

Keine Übelkeit ◆————————————◆ Schlimmste Form der Übelkeit

Tagebuch für den ersten Tag nach der Chemotherapie

Datum: _______________

____

Bitte die Schweregrade der Übelkeit folgendermaßen angeben:

Grad 1 (1): leichte Übelkeit (Übelkeit, Magenverstimmung, die beherrschbar ist und die Alltagsaktivitäten, wenn überhaupt, nur minimal beeinflusst)

Grad 2 (2): mittelschwere Übelkeit (stärkere Übelkeit, die manchmal mit dem Gefühl verbunden ist, erbrechen zu müssen, jedoch ohne tatsächliches Erbrechen, Alltagsaktivitäten wie Arbeiten, Essen, Trinken, Zubereiten einer Mahlzeit,… sind erschwert

Grad 3 (3): starke Übelkeit (sehr starke Übelkeit oder Gefühl, dass man sich fast dauernd übergeben muss, wobei man die Alltagsaktivitäten nicht ausführen kann)

	0 Uhr	1	2	3	4	5	6	7	8	9	10	11	12	13	14	15	16	17	18	19	20	21	22	23
Übelkeit (bitte Schweregrad angeben)																								
Brechreiz																								
Erbrechen																								
Einnahme von anderen Medikamenten																								
Gegessen																								
Getrunken																								

Falls Sie zusätzlich zur Studienmedikation Medikamente gegen Übelkeit und Erbrechen/Brechreiz genommen haben geben Sie bitte hier die Uhrzeit der Einnahme, Namen des Präparates, Dosierung, Anzahl der Einnahmen und die Darreichungsform des Medikaments an.
Bitte tragen Sie je Zeile nur ein Medikament ein. Tragen Sie dann in die oben stehende Tabelle bei der entsprechenden Uhrzeit die Nummer ein.

Nummer	Uhrzeit	Name des Medikaments	Dosierung	Anzahl der Einnahme	Darreichungsform
1					☐ Tablette<br>☐ Zäpfchen<br>☐ Tropfen
2					☐ Tablette<br>☐ Zäpfchen<br>☐ Tropfen
3					☐ Tablette<br>☐ Zäpfchen<br>☐ Tropfen
4					☐ Tablette<br>☐ Zäpfchen<br>☐ Tropfen
5					☐ Tablette<br>☐ Zäpfchen<br>☐ Tropfen
6					☐ Tablette<br>☐ Zäpfchen<br>☐ Tropfen
7					☐ Tablette<br>☐ Zäpfchen<br>☐ Tropfen
8					☐ Tablette

					☐ Zäpfchen<br>☐ Tropfen

Am Ende des Tages: Bitte beurteilen Sie den schlimmsten Grad der Übelkeit, der bei Ihnen seit dem letzten Eintrag aufgetreten ist, indem Sie einen senkrechten Strich (|) auf der nachstehenden Linie eintragen.

Keine Übelkeit ◆————————————◆ Schlimmste Form der Übelkeit

SUCCESS C

Tagebuch für den zweiten Tag nach Chemotherapie

Datum: ________________

Bitte die Schweregrade der Übelkeit folgendermaßen angeben:

Grad 1 (1): leichte Übelkeit (Übelkeit, Magenverstimmung, die beherrschbar ist und die Alltagsaktivitäten, wenn überhaupt, nur minimal beeinflusst)

Grad 2 (2): mittelschwere Übelkeit (stärkere Übelkeit, die manchmal mit dem Gefühl verbunden ist, erbrechen zu müssen, jedoch ohne tatsächliches Erbrechen, Alltagsaktivitäten wie Arbeiten, Essen, Trinken, Zubereiten einer Mahlzeit,… sind erschwert

Grad 3 (3): starke Übelkeit (sehr starke Übelkeit oder Gefühl, dass man sich fast dauernd übergeben muss, wobei man die Alltagsaktivitäten nicht ausführen kann)

	0 Uhr	1	2	3	4	5	6	7	8	9	10	11	12	13	14	15	16	17	18	19	20	21	22	23
Übelkeit (bitte Schweregrad angeben)																								
Brechreiz																								
Erbrechen																								
Einnahme von anderen Medikamenten																								
Gegessen																								
Getrunken																								

Falls Sie zusätzlich zur Studienmedikation Medikamente gegen Übelkeit und Erbrechen/Brechreiz genommen haben geben Sie bitte hier die Uhrzeit der Einnahme, Namen des Präparates, Dosierung, Anzahl der Einnahmen und die Darreichungsform des Medikaments an.
Bitte tragen Sie je Zeile nur ein Medikament ein. Tragen Sie dann in die oben stehende Tabelle bei der entsprechenden Uhrzeit die Nummer ein.

Nummer	Uhrzeit	Name des Medikaments	Dosierung	Anzahl der Einnahme	Darreichungsform
1					☐ Tablette<br>☐ Zäpfchen<br>☐ Tropfen
2					☐ Tablette<br>☐ Zäpfchen<br>☐ Tropfen
3					☐ Tablette<br>☐ Zäpfchen<br>☐ Tropfen
4					☐ Tablette<br>☐ Zäpfchen<br>☐ Tropfen
5					☐ Tablette<br>☐ Zäpfchen<br>☐ Tropfen
6					☐ Tablette<br>☐ Zäpfchen<br>☐ Tropfen
7					☐ Tablette<br>☐ Zäpfchen<br>☐ Tropfen
8					☐ Tablette

					☐ Zäpfchen<br>☐ Tropfen

Am Ende des Tages: Bitte beurteilen Sie den schlimmsten Grad der Übelkeit, der bei Ihnen seit dem letzten Eintrag aufgetreten ist, indem Sie einen senkrechten Strich (|) auf der nachstehenden Linie eintragen.

Keine Übelkeit ◆————————◆ Schlimmste Form der Übelkeit

SUCCESS C

Tagebuch für den dritten Tag nach der Chemotherapie

Datum: ________________

Bitte die Schweregrade der Übelkeit folgendermaßen angeben:

Grad 1 (1): leichte Übelkeit (Übelkeit, Magenverstimmung, die beherrschbar ist und die Alltagsaktivitäten, wenn überhaupt, nur minimal beeinflusst)

Grad 2 (2): mittelschwere Übelkeit (stärkere Übelkeit, die manchmal mit dem Gefühl verbunden ist, erbrechen zu müssen, jedoch ohne tatsächliches Erbrechen, Alltagsaktivitäten wie Arbeiten, Essen, Trinken, Zubereiten einer Mahlzeit,… sind erschwert

Grad 3 (3): starke Übelkeit (sehr starke Übelkeit oder Gefühl, dass man sich fast dauernd übergeben muss, wobei man die Alltagsaktivitäten nicht ausführen kann)

	0 Uhr	1	2	3	4	5	6	7	8	9	10	11	12	13	14	15	16	17	18	19	20	21	22	23
Übelkeit (bitte Schweregrad angeben)																								
Brechreiz																								
Erbrechen																								
Einnahme von anderen Medikamenten																								
Gegessen																								
Getrunken																								

Falls Sie zusätzlich zur Studienmedikation Medikamente gegen Übelkeit und Erbrechen/Brechreiz genommen haben geben Sie bitte hier die Uhrzeit der Einnahme, Namen des Präparates, Dosierung, Anzahl der Einnahmen und die Darreichungsform des Medikaments an.
Bitte tragen Sie je Zeile nur ein Medikament ein. Tragen Sie dann in die oben stehende Tabelle bei der entsprechenden Uhrzeit die Nummer ein.

Nummer	Uhrzeit	Name des Medikaments	Dosierung	Anzahl der Einnahme	Darreichungsform
1					☐ Tablette<br>☐ Zäpfchen<br>☐ Tropfen
2					☐ Tablette<br>☐ Zäpfchen<br>☐ Tropfen
3					☐ Tablette<br>☐ Zäpfchen<br>☐ Tropfen
4					☐ Tablette<br>☐ Zäpfchen<br>☐ Tropfen
5					☐ Tablette<br>☐ Zäpfchen<br>☐ Tropfen
6					☐ Tablette<br>☐ Zäpfchen<br>☐ Tropfen
7					☐ Tablette<br>☐ Zäpfchen<br>☐ Tropfen
8					☐ Tablette

					☐ Zäpfchen<br>☐ Tropfen

Bitte beurteilen Sie den schlimmsten Grad der Übelkeit, der bei Ihnen seit dem letzten Eintrag aufgetreten ist, indem Sie einen senkrechten Strich (|) auf der nachstehenden Linie eintragen.

Keine Übelkeit ◆————————◆ Schlimmste Form der Übelkeit

SUCCESS C

Tagebuch für den vierten Tag nach der Chemotherapie

Datum: _______________

Bitte die Schweregrade der Übelkeit folgendermaßen angeben:

Grad 1 (1): leichte Übelkeit (Übelkeit, Magenverstimmung, die beherrschbar ist und die Alltagsaktivitäten, wenn überhaupt, nur minimal beeinflusst)

Grad 2 (2): mittelschwere Übelkeit (stärkere Übelkeit, die manchmal mit dem Gefühl verbunden ist, erbrechen zu müssen, jedoch ohne tatsächliches Erbrechen, Alltagsaktivitäten wie Arbeiten, Essen, Trinken, Zubereiten einer Mahlzeit,… sind erschwert

Grad 3 (3): starke Übelkeit (sehr starke Übelkeit oder Gefühl, dass man sich fast dauernd übergeben muss, wobei man die Alltagsaktivitäten nicht ausführen kann)

	0 Uhr	1	2	3	4	5	6	7	8	9	10	11	12	13	14	15	16	17	18	19	20	21	22	23
Übelkeit (bitte Schweregrad angeben)																								
Brechreiz																								
Erbrechen																								
Einnahme von anderen Medikamenten																								
Gegessen																								
Getrunken																								

Falls Sie zusätzlich zur Studienmedikation Medikamente gegen Übelkeit und Erbrechen/Brechreiz genommen haben geben Sie bitte hier die Uhrzeit der Einnahme, Namen des Präparates, Dosierung, Anzahl der Einnahmen und die Darreichungsform des Medikaments an.
Bitte tragen Sie je Zeile nur ein Medikament ein. Tragen Sie dann in die oben stehende Tabelle bei der entsprechenden Uhrzeit die Nummer ein.

Nummer	Uhrzeit	Name des Medikaments	Dosierung	Anzahl der Einnahme	Darreichungsform
1					☐ Tablette<br>☐ Zäpfchen<br>☐ Tropfen
2					☐ Tablette<br>☐ Zäpfchen<br>☐ Tropfen
3					☐ Tablette<br>☐ Zäpfchen<br>☐ Tropfen
4					☐ Tablette<br>☐ Zäpfchen<br>☐ Tropfen
5					☐ Tablette<br>☐ Zäpfchen<br>☐ Tropfen
6					☐ Tablette<br>☐ Zäpfchen<br>☐ Tropfen
7					☐ Tablette<br>☐ Zäpfchen<br>☐ Tropfen
8					☐ Tablette

					☐ Zäpfchen<br>☐ Tropfen

Bitte beurteilen Sie den schlimmsten Grad der Übelkeit, der bei Ihnen seit dem letzten Eintrag aufgetreten ist, indem Sie einen senkrechten Strich (|) auf der nachstehenden Linie eintragen.

◆————————————————————◆

Keine Übelkeit | Schlimmste Form der Übelkeit

Tagebuch für den fünften Tag nach der Chemotherapie

Datum: ________________

Bitte die Schweregrade der Übelkeit folgendermaßen angeben:

Grad 1 (1): leichte Übelkeit (Übelkeit, Magenverstimmung, die beherrschbar ist und die Alltagsaktivitäten, wenn überhaupt, nur minimal beeinflusst)

Grad 2 (2): mittelschwere Übelkeit (stärkere Übelkeit, die manchmal mit dem Gefühl verbunden ist, erbrechen zu müssen, jedoch ohne tatsächliches Erbrechen, Alltagsaktivitäten wie Arbeiten, Essen, Trinken, Zubereiten einer Mahlzeit,… sind erschwert

Grad 3 (3): starke Übelkeit (sehr starke Übelkeit oder Gefühl, dass man sich fast dauernd übergeben muss, wobei man die Alltagsaktivitäten nicht ausführen kann)

	0 Uhr	1	2	3	4	5	6	7	8	9	10	11	12	13	14	15	16	17	18	19	20	21	22	23
Übelkeit (bitte Schweregrad angeben)																								
Brechreiz																								
Erbrechen																								
Einnahme von anderen Medikamenten																								
Gegessen																								
Getrunken																								

Falls Sie zusätzlich zur Studienmedikation Medikamente gegen Übelkeit und Erbrechen/Brechreiz genommen haben geben Sie bitte hier die Uhrzeit der Einnahme, Namen des Präparates, Dosierung, Anzahl der Einnahmen und die Darreichungsform des Medikaments an.
Bitte tragen Sie je Zeile nur ein Medikament ein. Tragen Sie dann in die oben stehende Tabelle bei der entsprechenden Uhrzeit die Nummer ein.

Nummer	Uhrzeit	Name des Medikaments	Dosierung	Anzahl der Einnahme	Darreichungsform
1					☐ Tablette<br>☐ Zäpfchen<br>☐ Tropfen
2					☐ Tablette<br>☐ Zäpfchen<br>☐ Tropfen
3					☐ Tablette<br>☐ Zäpfchen<br>☐ Tropfen
4					☐ Tablette<br>☐ Zäpfchen<br>☐ Tropfen
5					☐ Tablette<br>☐ Zäpfchen<br>☐ Tropfen
6					☐ Tablette<br>☐ Zäpfchen<br>☐ Tropfen
7					☐ Tablette<br>☐ Zäpfchen<br>☐ Tropfen
8					☐ Tablette

					☐ Zäpfchen<br>☐ Tropfen

Wichtig: Bitte etwa 120 Stunden nach der Chemotherapie eintragen, am Ende von Tag 5: Bitte beurteilen Sie den schlimmsten Grad der Übelkeit, der bei Ihnen seit dem letzten Eintrag aufgetreten ist, indem Sie einen senkrechten Strich (|) auf der nachstehenden Linie eintragen.

◆————————————————————◆

Keine Übelkeit — Schlimmste Form der Übelkeit

8.7 Eastern Cooperative Oncology Group (ECOG) Performance Status

Activity Status	Description
**0**	Asymptomatic, fully active, and able to carry on all predisease performance without restrictions
**1**	Symptomatic, fully ambulatory but restricted in physically strenuous activity and able to carry out performance of a light or sedentary nature, eg, light housework, office work
**2**	Symptomatic, ambulatory and capable of all self-care but unable to carry out any work activities. Up and about more than 50 % of waking hours: in bed less than 50 % of day
**3**	Symptomatic, capable on only limited self-care, confined to bed or chair more than 50 % of waking hours, but not bedridden
**4**	Completely diabled. Cannot carry on any self-care. Totally bedridden
**5**	Dead
