# Supplementary material for: Impact of pre-chemotherapy food intake on chemotherapy-induced nausea and vomiting in patients with early breast cancer: a subanalysis of the randomized SUCCESS C trial
Source: Support Care Cancer. 2026 Nov 13;34(12):1223. doi: 10.1007/s00520-026-11437-9 (PMC13768299; doi:10.1007/s00520-026-11437-9)
Supplement: Supplementary file 2 [file 520_2026_11437_MOESM1_ESM.docx]

**Supplemental information**

**Impact of Pre-Chemotherapy Food Intake on Chemotherapy-Induced Nausea and Vomiting in Patients with Early Breast Cancer: A Subanalysis of the randomized SUCCESS C Trial**

**Henning Schäffler^2^, Manuel Hörner****^1^, Lothar Häberle^1,3^, Chloë Goossens^1^, Kerstin Pfister^2^, Elena Leinert^2^, Kristina Veselinovic^2^, Sara Y. Brucker^4^, Uwe Köhler^5^, Georg Heinrich^6^, Andreas Schneeweiss^7^, Matthias W. Beckmann^1^, Peter A. Fasching^1^, Wolfgang W. Janni^2^, Brigitte Rack^2^, Sabine Heublein^2^, Philipp Ziegler^1^**

^1^ Department of Gynecology and Obstetrics, Universitätsklinikum Erlangen, Comprehensive Cancer Center Erlangen-EMN, Friedrich-Alexander-Universität Erlangen-Nürnberg, Erlangen, Germany

^2^ Department of Gynecology and Obstetrics, Ulm University Hospital, Ulm, Germany

^3^ Biostatistics Unit, Department of Gynecology and Obstetrics, Universitätsklinikum Erlangen, Erlangen, Germany

^4^ Department of Women’s Health, Tuebingen University, Tübingen, Germany

^5^ Department of Gynecology and Obstetrics, Klinikum St. Georg, Leipzig, Germany

^6^ Schwerpunktpraxis der Gynäkologie und Onkologie Fürstenwalde, Klinikum Offenbach, Fürstenwalde/Spree, Germany

^7^ Gynecologic Oncology, National Center for Tumor Diseases (NCT), University of Heidelberg and German Cancer Research Center (DKFZ), Heidelberg, Germany

Supplemental table 1. Without pre-chemotherapy food intake vs. Pre-chemotherapy food intake in TC cycle 1-3 for patient and CINV characteristics, showing frequency and percentage.

| **Characteristic** |  | **Cycle 1** | | **Cycle 2** | | **Cycle 3** | |
| --- | --- | --- | --- | --- | --- | --- | --- |
|  |  | **without pre-chemotherapy food intake (N=420)** | **Pre-chemotherapy food intake (N=288)** | **without pre-chemotherapy food intake (N=255)** | **Pre-chemotherapy food intake (N=444)** | **without pre-chemotherapy food intake (N=221)** | **Pre-chemotherapy food intake (N=475)** |
| Age | Age < 50 | 136 (58.1%) | 98 (41.9%) | 78 (33.5%) | 155 (66.5%) | 74 (31.8%) | 159 (68.2%) |
|  | 50 <= Age < 65 | 201 (60.5%) | 131 (39.5%) | 127 (38.5%) | 203 (61.5%) | 103 (31.7%) | 222 (68.3%) |
|  | Age >= 65 | 83 (58.5%) | 59 (41.5%) | 50 (36.8%) | 86 (63.2%) | 44 (31.9%) | 94 (68.1%) |
| Alcohol per week | never | 163 (58.0%) | 118 (42.0%) | 107 (37.5%) | 178 (62.5%) | 87 (31.4%) | 190 (68.6%) |
|  | 1-2x | 179 (61.9%) | 110 (38.1%) | 98 (35.1%) | 181 (64.9%) | 84 (29.8%) | 198 (70.2%) |
|  | 3-7x | 51 (60.7%) | 33 (39.3%) | 35 (41.7%) | 49 (58.3%) | 31 (36.9%) | 53 (63.1%) |
|  | NA | 27 | 27 | 15 | 36 | 19 | 34 |
| Smoking | non-smoker | 347 (58.0%) | 251 (42.0%) | 212 (35.8%) | 380 (64.2%) | 183 (31.2%) | 403 (68.8%) |
|  | smoker | 41 (67.2%) | 20 (32.8%) | 25 (41.0%) | 36 (59.0%) | 26 (40.6%) | 38 (59.4%) |
|  | NA | 32 | 17 | 18 | 28 | 12 | 34 |
| Body mass index | overweight/adipositas | 220 (57.4%) | 163 (42.6%) | 144 (37.5%) | 240 (62.5%) | 118 (31.1%) | 262 (68.9%) |
|  | underweight/normal | 200 (61.5%) | 125 (38.5%) | 111 (35.2%) | 204 (64.8%) | 103 (32.6%) | 213 (67.4%) |
| Pre-cycle emetic episode | No | - | - | 178 (34.9%) | 332 (65.1%) | 160 (30.1%) | 371 (69.9%) |
|  | Yes | - | - | 76 (40.9%) | 110 (59.1%) | 58 (36.2%) | 102 (63.7%) |
|  | NA | - | - | 1 | 2 | 3 | 2 |
| Pre-cycle nausea episode | No | - | - | 107 (37.3%) | 180 (62.7%) | 107 (31.4%) | 234 (68.6%) |
|  | Yes | - | - | 147 (35.9%) | 262 (64.1%) | 111 (31.7%) | 239 (68.3%) |
|  | NA | - | - | 1 | 2 | 3 | 2 |
| Prophylactic antiemetic medication | Aprepitant | 14 (40.0%) | 21 (60.0%) | 9 (24.3%) | 28 (75.7%) | 10 (27.8%) | 26 (72.2%) |
|  | Dexamethason | 61 (50.4%) | 60 (49.6%) | 33 (28.9%) | 81 (71.1%) | 33 (31.7%) | 71 (68.3%) |
|  | Ondansetron or eq. | 9 (40.9%) | 13 (59.1%) | 14 (42.4%) | 19 (57.6%) | 12 (40.0%) | 18 (60.0%) |

Supplemental table 2. Without pre-chemotherapy food intake vs. Pre-chemotherapy food intake in FEC cycle 1-3 for patient and CINV characteristics, showing frequency and percentage.

| **Characteristic** |  | **Cycle 1** | | **Cycle 2** | | **Cycle 3** | |
| --- | --- | --- | --- | --- | --- | --- | --- |
|  |  | **without pre-chemotherapy food intake (N=488)** | **Pre-chemotherapy food intake (N=228)** | **without pre-chemotherapy food intake (N=344)** | **Pre-chemotherapy food intake (N=380)** | **without pre-chemotherapy food intake (N=306)** | **Pre-chemotherapy food intake (N=399)** |
| Age | Age < 50 | 161 (69.1%) | 72 (30.9%) | 121 (50.8%) | 117 (49.2%) | 102 (44.2%) | 129 (55.8%) |
|  | 50 <= Age < 65 | 241 (66.0%) | 124 (34.0%) | 167 (45.5%) | 200 (54.5%) | 154 (42.7%) | 207 (57.3%) |
|  | Age >= 65 | 86 (72.9%) | 32 (27.1%) | 56 (47.1%) | 63 (52.9%) | 50 (44.2%) | 63 (55.8%) |
| Alcohol per week | never | 199 (67.0%) | 98 (33.0%) | 153 (51.2%) | 146 (48.8%) | 135 (45.8%) | 160 (54.2%) |
|  | 1-2x | 210 (68.6%) | 96 (31.4%) | 140 (45.0%) | 171 (55.0%) | 121 (40.2%) | 180 (59.8%) |
|  | 3-7x | 51 (72.9%) | 19 (27.1%) | 33 (45.8%) | 39 (54.2%) | 29 (43.3%) | 38 (56.7%) |
|  | NA | 28 | 15 | 18 | 24 | 21 | 21 |
| Smoking | non-smoker | 399 (68.7%) | 182 (31.3%) | 281 (48.1%) | 303 (51.9%) | 245 (43.1%) | 323 (56.9%) |
|  | smoker | 50 (71.4%) | 20 (28.6%) | 37 (51.4%) | 35 (48.6%) | 36 (49.3%) | 37 (50.7%) |
|  | NA | 39 | 26 | 26 | 42 | 25 | 39 |
| Body mass index | overweight/adipositas | 262 (68.8%) | 119 (31.2%) | 182 (47.5%) | 201 (52.5%) | 170 (45.6%) | 203 (54.4%) |
|  | underweight/normal | 226 (67.5%) | 109 (32.5%) | 162 (47.5%) | 179 (52.5%) | 136 (41.0%) | 196 (59.0%) |
| Pre-cycle emetic episode | No | - | - | 181 (44.7%) | 224 (55.3%) | 176 (41.3%) | 250 (58.7%) |
|  | Yes | - | - | 157 (50.2%) | 156 (49.8%) | 129 (46.4%) | 149 (53.6%) |
|  | NA | - | - | 6 | 0 | 1 | 0 |
| Pre-cycle nausea episode | No | - | - | 70 (45.8%) | 83 (54.2%) | 84 (43.8%) | 108 (56.2%) |
|  | Yes | - | - | 268 (47.4%) | 297 (52.6%) | 221 (43.2%) | 291 (56.8%) |
|  | NA | - | - | 6 | 0 | 1 | 0 |
| Prophylactic antiemetic medication | Aprepitant | 55 (68.8%) | 25 (31.2%) | 36 (35.0%) | 67 (65.0%) | 35 (34.3%) | 67 (65.7%) |
|  | Dexamethason | 22 (53.7%) | 19 (46.3%) | 18 (37.5%) | 30 (62.5%) | 16 (44.4%) | 20 (55.6%) |
|  | Ondansetron or eq. | 24 (72.7%) | 9 (27.3%) | 19 (51.4%) | 18 (48.6%) | 15 (45.5%) | 18 (54.5%) |

Supplemental table 3. Median nausea-free times and nausea-free rates relative to chemo type, eating behaviour and chemo cycle.

| **Chemo**  **type** | **Eating behaviour** | **Chemo**  **cycle** | **Patients** | **Events** | **Median  nausea-free time in hours (95% CI)** | **12-hour  nausea-free rate (95% CI)** | **24-hour  nausea-free rate (95% CI)** | **48-hour nausea-free rate (95% CI)** | **120-hour**  **nausea-free rate**  **(95% CI)** |
| --- | --- | --- | --- | --- | --- | --- | --- | --- | --- |
| TC | without pre-chemotherapy food intake | 1 | 420 | 249 | 64.0 (44.0, 93.0) | 0.77 (0.73, 0.81) | 0.60 (0.55, 0.64) | 0.53 (0.49, 0.58) | 0.41 (0.36, 0.46) |
|  |  | 2 | 255 | 132 | 92.0 (53.0, NA) | 0.82 (0.77, 0.87) | 0.64 (0.58, 0.70) | 0.58 (0.52, 0.65) | 0.48 (0.42, 0.54) |
|  |  | 3 | 221 | 102 | NA (81.0, NA) | 0.80 (0.75, 0.86) | 0.70 (0.64, 0.77) | 0.61 (0.55, 0.68) | 0.53 (0.46, 0.60) |
| TC | Pre-chemotherapy food intake | 1 | 288 | 167 | 75.0 (48.0, 96.0) | 0.79 (0.74, 0.84) | 0.66 (0.60, 0.71) | 0.55 (0.50, 0.61) | 0.42 (0.37, 0.48) |
|  |  | 2 | 444 | 221 | NA (81.0, NA) | 0.86 (0.82, 0.89) | 0.72 (0.68, 0.76) | 0.64 (0.60, 0.69) | 0.50 (0.46, 0.55) |
|  |  | 3 | 475 | 214 | NA ( NA, NA) | 0.86 (0.83, 0.89) | 0.76 (0.72, 0.80) | 0.66 (0.62, 0.71) | 0.55 (0.51, 0.59) |
| FEC | without pre-chemotherapy food intake | 1 | 488 | 390 | 9.0 (8.0, 10.0) | 0.39 (0.35, 0.43) | 0.31 (0.27, 0.35) | 0.26 (0.22, 0.30) | 0.20 (0.16, 0.24) |
|  |  | 2 | 344 | 257 | 9.0 ( 8.0, 11.0) | 0.43 (0.38, 0.49) | 0.34 (0.29, 0.39) | 0.29 (0.24, 0.34) | 0.25 (0.20, 0.30) |
|  |  | 3 | 306 | 218 | 9.0 ( 8.0, 13.0) | 0.44 (0.39, 0.50) | 0.39 (0.34, 0.45) | 0.35 (0.30, 0.40) | 0.28 (0.23, 0.34) |
| FEC | Pre-chemotherapy food intake | 1 | 228 | 167 | 10.5 (9.0, 23.0) | 0.48 (0.42, 0.55) | 0.42 (0.36, 0.49) | 0.34 (0.28, 0.40) | 0.27 (0.21, 0.33) |
|  |  | 2 | 380 | 271 | 12.0 (10.0, 20.0) | 0.50 (0.45, 0.55) | 0.40 (0.36, 0.45) | 0.34 (0.30, 0.40) | 0.29 (0.24, 0.34) |
|  |  | 3 | 399 | 265 | 16.0 (10.0, 25.0) | 0.51 (0.47, 0.57) | 0.45 (0.40, 0.50) | 0.41 (0.37, 0.46) | 0.33 (0.29, 0.38) |

CI confidence interval

Supplemental table 4. Median emesis-free rates relative to chemo type, eating behaviour and chemo cycle^[1]^.

| **Chemo**  **type** | **Eating behaviour** | **Chemo**  **cycle** | **Patients** | **Events** | **12-hour  emesis-free rate (95% CI)** | **24-hour  emesis-free rate (95% CI)** | **48-hour emesis-free rate (95% CI)** | **120-hour**  **emesis-free rate**  **(95% CI)** |
| --- | --- | --- | --- | --- | --- | --- | --- | --- |
| TC | without pre-chemotherapy food intake | 1 | 420 | 120 | 0.91 (0.88, 0.94) | 0.84 (0.80, 0.87) | 0.81 (0.77, 0.85) | 0.71 (0.67, 0.76) |
|  |  | 2 | 255 | 76 | 0.93 (0.89, 0.96) | 0.82 (0.77, 0.87) | 0.78 (0.73, 0.84) | 0.70 (0.64, 0.76) |
|  |  | 3 | 221 | 55 | 0.91 (0.88, 0.95) | 0.84 (0.79, 0.89) | 0.81 (0.76, 0.87) | 0.74 (0.69, 0.81) |
| TC | Pre-chemotherapy food intake | 1 | 288 | 67 | 0.92 (0.89, 0.95) | 0.88 (0.85, 0.92) | 0.86 (0.83, 0.90) | 0.77 (0.72, 0.82) |
|  |  | 2 | 444 | 87 | 0.94 (0.92, 0.96) | 0.90 (0.87, 0.93) | 0.87 (0.84, 0.90) | 0.80 (0.77, 0.84) |
|  |  | 3 | 475 | 102 | 0.95 (0.93, 0.97) | 0.91 (0.89, 0.94) | 0.87 (0.84, 0.90) | 0.78 (0.75, 0.82) |
| FEC | without pre-chemotherapy food intake | 1 | 488 | 216 | 0.69 (0.65, 0.73) | 0.63 (0.59, 0.67) | 0.60 (0.56, 0.64) | 0.56 (0.51, 0.60) |
|  |  | 2 | 344 | 152 | 0.73 (0.68, 0.78) | 0.66 (0.61, 0.71) | 0.61 (0.56, 0.66) | 0.55 (0.50, 0.61) |
|  |  | 3 | 306 | 129 | 0.70 (0.65, 0.75) | 0.66 (0.61, 0.72) | 0.63 (0.57, 0.68) | 0.57 (0.52, 0.63) |
| FEC | Pre-chemotherapy food intake | 1 | 228 | 91 | 0.75 (0.70, 0.81) | 0.72 (0.66, 0.78) | 0.67 (0.61, 0.73) | 0.60 (0.54, 0.67) |
|  |  | 2 | 380 | 132 | 0.79 (0.75, 0.83) | 0.74 (0.70, 0.79) | 0.71 (0.67, 0.76) | 0.65 (0.61, 0.70) |
|  |  | 3 | 399 | 138 | 0.79 (0.75, 0.83) | 0.74 (0.69, 0.78) | 0.71 (0.67, 0.76) | 0.65 (0.61, 0.70) |

CI confidence interval

1. Median emesis-free time was not reached within 120h observation period.
